# Self-navigated 3D diffusion MRI using an optimized CAIPI sampling and structured low-rank reconstruction estimated navigator

**DOI:** 10.1109/TMI.2024.3454994

**Published:** 2024-09-06

**Authors:** Ziyu Li, Karla L. Miller, Xi Chen, Mark Chiew, Wenchuan Wu

**Affiliations:** Wellcome Centre for Integrative Neuroimaging, FMRIB, Nuffield Department of Clinical Neurosciences, University of Oxford, Oxford, UK; Wellcome Centre for Integrative Neuroimaging, FMRIB, Nuffield Department of Clinical Neurosciences, University of Oxford, Oxford, UK; Wellcome Centre for Integrative Neuroimaging, FMRIB, Nuffield Department of Clinical Neurosciences, University of Oxford, Oxford, UK; Department of Radiological Sciences, David Geffen School of Medicine at UCLA, Los Angeles, California, USA; Wellcome Centre for Integrative Neuroimaging, FMRIB, Nuffield Department of Clinical Neurosciences, University of Oxford, Oxford, UK; Physical Sciences, Sunnybrook Research Institute and Department of Medical Biophysics, University of Toronto, Toronto, Canada; Wellcome Centre for Integrative Neuroimaging, FMRIB, Nuffield Department of Clinical Neurosciences, University of Oxford, Oxford, UK

**Keywords:** 3D multi-slab imaging, self-navigation, motion correction, phase error correction, sampling optimization

## Abstract

3D multi-slab acquisitions are an appealing approach for diffusion MRI because they are compatible with the imaging regime delivering optimal SNR efficiency. In conventional 3D multi-slab imaging, shot-to-shot phase variations caused by motion pose challenges due to the use of multi-shot k-space acquisition. Navigator acquisition after each imaging echo is typically employed to correct phase variations, which prolongs scan time and increases the specific absorption rate (SAR). The aim of this study is to develop a highly efficient, self-navigated method to correct for phase variations in 3D multi-slab diffusion MRI without explicitly acquiring navigators. The sampling of each shot is carefully designed to intersect with the central kz=0 plane of each slab, and the multi-shot sampling is optimized for self-navigation performance while retaining decent reconstruction quality. The kz=0 intersections from all shots are jointly used to reconstruct a 2D phase map for each shot using a structured low-rank constrained reconstruction that leverages the redundancy in shot and coil dimensions. The phase maps are used to eliminate the shot-to-shot phase inconsistency in the final 3D multi-shot reconstruction. We demonstrate the method’s efficacy using retrospective simulations and prospectively acquired in-vivo experiments at 1.22 mm and 1.09 mm isotropic resolutions. Compared to conventional navigated 3D multi-slab imaging, the proposed self-navigated method achieves comparable image quality while shortening the scan time by 31.7% and improving the SNR efficiency by 15.5%. The proposed method produces comparable quality of DTI and white matter tractography to conventional navigated 3D multi-slab acquisition with a much shorter scan time.

## I Introduction

High-resolution diffusion MRI can provide detailed information about tissue microstructure and accurate representation of intricate fiber arrangements [1, 2]. However, the effectiveness of diffusion MRI is limited by its inherently low SNR, which further decreases as the resolution increases.

3D multi-slab imaging is a promising approach for high-resolution diffusion MRI due to its ability to achieve short TR=1-2s [3–6] for optimal SNR efficiency for spin-echo based diffusion MRI. This technique divides the entire imaging volume into multiple thin slabs with 10-20 slices per slab, typically employing a 3D multi-shot echo-planar imaging (EPI) trajectory for high efficiency [3–6]. However, this method is sensitive to motion-induced shot-to-shot phase variations that degrade image quality when combining data from different shots [7, 8].

Conventional methods to correct the phase inconsistency in multi-shot diffusion MRI require navigators [3–8]. The navigators are incorporated into a model-based reconstruction to correct phase errors [7]. Previous studies have shown that the motion-induced phase within each 3D slab can be approximated by a 2D navigator if the slabs are sufficiently thin (i.e., <2 cm) [3–6]. This 2D navigator necessitates an additional spin echo, leading to an extended TR and increasing the scan time by 25%-50% [5, 9]. Furthermore, the inclusion of another RF refocusing pulse increases the specific absorption rate (SAR).

Several studies have investigated the feasibility of navigator-free 2D multi-shot diffusion imaging. Multiplexed sensitivity-encoding (MUSE) reconstructs a 2D phase map from each under-sampled shot [10]. Despite the relatively high under-sampling factor per shot, clean phase maps can be reconstructed by exploiting the smoothness of motion-induced phase. Methods avoiding an explicit phase map have also been proposed [11, 12], which leverage the magnitude consistency between shots to create a structured Hankel matrix and estimate the missing data in each shot using structured low-rank (SLR) matrix completion [13].

Extending these methods for navigator-free 3D multi-slab diffusion imaging is intrinsically challenging. Specifically, to extend MUSE to 3D requires estimating a 2D phase map from each shot to capture in-plane phase variations. However, for conventional 3D Cartesian EPI (e.g., [3–5]), shots covering peripheral kz planes encode high spatial frequency information, and estimating in-plane smooth phase variations is challenging. Additionally, in conventional 3D multi-slab diffusion imaging, each shot is effectively subjected to a high under-sampling factor with little redundancy between shots, which significantly hampers the feasibility of extending 2D SLR approach to 3D. Therefore, simplistically applying the 2D SLR approach to 3D multi-slab diffusion imaging might be impractical.

In this study, we present a novel acquisition and reconstruction framework for self-navigated 3D multi-slab diffusion MRI. We propose a new 3D EPI sampling pattern that enables self-navigation with minimized k-space gaps in the shot-combined sampling and minimized overlapping between shots. A k-space based SLR constrained reconstruction is leveraged to jointly exploit data redundancy across shots and coils to reconstruct high-quality phase maps. Our multi-shot reconstruction incorporates phase error correction to calculate the final image. The proposed method’s efficacy is evaluated through in-vivo experiments on a 7T scanner. The results demonstrate comparable image quality and significantly improved SNR efficiency compared to navigator-based methods. The resulting whole-brain diffusion tensor imaging (DTI) and tractography results highlight our proposed method’s potential to enable high-resolution 3D multi-slab diffusion imaging with improved time efficiency.

## II Theory

### A Review of 3D multi-slab diffusion imaging

**Fig. 1:**
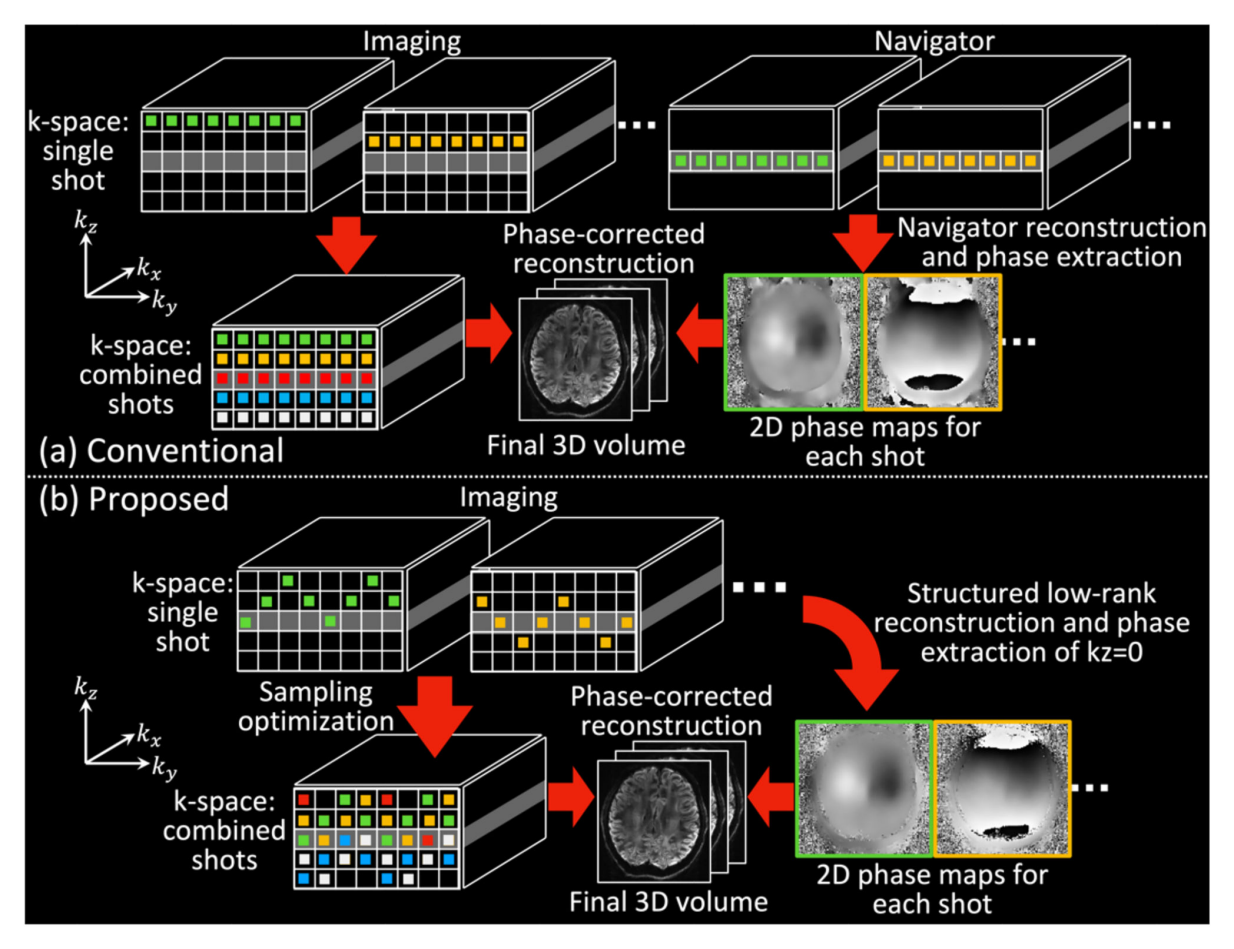
Comparison between conventional navigated 3D multi-slab imaging (a) and the proposed self-navigated imaging framework (b). In the conventional approach (a), phase information is obtained from a separately acquired 2D navigator. In contrast, the proposed method (b) extracts shot phase directly from the imaging data using a novel acquisition and reconstruction framework, eliminating the need for acquiring a separate navigator.

3D multi-slab diffusion imaging uses multi-shot EPI to encode a 3D k-space for each slab. The acquired k-space signal for the *j*^*th*^ shot can be represented as:

(1) $$y\left(k_{j}\right)=\int \rho (r)\phi_{d_{j}}(r)e^{-i2\pi k_{j}^{T}r}dr+n(k_{j}),$$

where *y* is the diffusion k-space data, ***k***_***j***_ is the k-space sampling positions for the *j*^*th*^ shot, *ρ* is the phase error-free 3D image signal, ***r*** is position in the image space, $\phi_{d_{j}}=e^{i\psi_{j}}$, and *ψ*_*j*_ is the non-diffusive motion induced phase in shot *j*, *j* ∈ [1, *N*_*shot*_], where *N*_*shot*_ denotes the total numbers of shots. In conventional 3D multi-slab diffusion imaging, each shot covers a kz plane using a single EPI readout for efficient data acquisition (Fig. 1a), and *N*_*shot*_ equals to the number of encoded *k*_z_ planes $\left(N_{k_{z}}\right)$. *n* is the additive Gaussian noise. The signal formulation described in Eq. 1 can be extended to the multi-coil case and represented in a matrix form as:

(2) $$y_{j}={ℳ}_{j}F\phi_{d_{j}}X+n_{j},$$

where *X* is the matrix representation of the multi-coil phase error-free 3D image volume, y_*j*_ is the k-space sampling matrix of shot *j*, and *F* is the Fourier Transform.

Non-diffusive motions during diffusion encoding introduced shot-dependent phase errors *ϕ*_*d*_ can lead to significant image corruptions if not corrected. Therefore, accurate information of phase errors $\phi_{d_{j}}$ is necessary for the reconstruction of *X*.

In conventional 3D multi-slab diffusion imaging, a 2D navigator is acquired at *k*_*z*_ = 0 for each shot using an extra refocusing RF pulse (i.e., the navigator samples the secondary spin echo) and the phase of the navigator is used as an estimation of motion induced phase errors (Fig. 1a). However, navigator acquisition suffers from several drawbacks, including prolonged scan time, increased SAR associated with a second spin echo, and reduced SNR efficiency.

### B Review of SLR reconstruction for 2D navigator-free diffusion imaging

The 2D SLR reconstruction (e.g., MUSSELS [11, 12, 14]) leverages the data redundancy across different shots to effectively restore missing data. Consequently, it enables accurate estimation of magnitude and phase information for each shot without the need for explicitly acquired phase maps.

MUSSELS assumes different shots share the same underlying magnitude image m despite their different phases:

(3) $$m=x_{j_{1}}\Phi_{j_{1}}^{H}=x_{j_{2}}\Phi_{j_{2}}^{H},\;j_{1},j_{2}\in \left[1,N_{shot}\right],$$

where $x_{j_{1}}$ and $x_{j_{2}}$ are the complex image for shot *j*_1_ and *j*_2_, respectively, Φ = *ϕ*_*d*_*ϕ*_*c*_ includes the diffusion-related phase *ϕ*_*d*_ and coil-related phase *ϕ*_*c*_, and Φ^*H*^ denotes the conjugate of Φ. This leads to the establishment of an annihilation relation in both the image domain (Eq. 4) and the Fourier domain (Eq. 5):

(4) $$x_{j_{1}}\Phi_{j_{2}}-x_{j_{2}}\Phi_{j_{1}}=0,\;j_{1},j_{2}\in \left[1,N_{shot\;}\right],$$

(5) $${\hat{x}}_{j_{1}}*{\hat{\Phi }}_{j_{2}}-{\hat{x}}_{j_{2}}*{\hat{\Phi }}_{j_{1}}=0,\;j_{1},j_{2}\in \left[1,N_{shot\;}\right]$$

where $\hat{x}$ and $\hat{\Phi }$ denotes the Fourier Transform of *x* and Φ, respectively, and * denotes the convolution operation. As Φ is typically smooth in image space, $\hat{\Phi }$ should be support limited in the Fourier domain. Utilizing the block-Hankel matrix formulation $H_{1}(\hat{x})$ detailed in previous work [11], Eq. 5 can be expressed in a matrix form:

(6) $$\left[\begin{array}{c} H_{1}\left({\hat{x}}_{j_{1}}\right)H_{1}\left({\hat{x}}_{j_{2}}\right) \end{array}\right]\left[\begin{array}{c} vec\left({\hat{\Phi }}_{j_{2}}\right)\\ -vec\left({\hat{\Phi }}_{j_{1}}\right) \end{array}\right]=0,\;j_{1},j_{2}\in \left[1,N_{shot}\right].$$

This relation holds for all pairs of shots as they are all assumed to share the same magnitude image, Thus, the structured matrix

(7) $$H(\hat{x})=\left[\begin{array}{c} H_{1}\left({\hat{x}}_{1}\right)H_{1}\left({\hat{x}}_{2}\right)\ldots H_{1}\left({\hat{x}}_{N_{shot}}\right) \end{array}\right]$$

exhibits a low-rank property due to the existence of a non-trivial null space $P(H(\hat{x})P=0)$:

(8) $$\text{P}=\left[\begin{array}{cccc} {\hat{\Phi }}_{2} & 0 & 0 & {\hat{\Phi }}_{3}\\ -{\hat{\Phi }}_{1} & {\hat{\Phi }}_{3} & 0 & 0\\ 0 & -{\hat{\Phi }}_{2} & 0 & -{\hat{\Phi }}_{1}\\ \vdots & \vdots & \vdots & \vdots \\ 0 & 0 & {\hat{\Phi }}_{N_{shot\;}} & 0\\ 0 & 0 & -{\hat{\Phi }}_{N_{shot}-1} & 0 \end{array}\right].$$

The 2D SLR reconstruction enforces the low-rankness of $H(\hat{x})$ by minimizing $||H(\hat{x})|{|}_{*}$ during iterative reconstruction to facilitate high-fidelity navigator-free 2D diffusion imaging.

However, the extension of 2D SLR reconstruction to 3D multi-slab diffusion imaging presents notable challenges. While previous approaches, such as gSlider [15, 16], have effectively applied SLR to high-resolution multi-shot diffusion imaging, they essentially reconstructed RF-encoded 2D k-space. Our work focuses on leveraging SLR for 3D k-space reconstruction. A direct extension to 3D SLR (by substituting 2D images in Eq. 3 with 3D volumes) requires reconstructing an entire 3D volume from a single shot, which undergoes exceptionally high under-sampling factor for 3D multi-slab imaging. For instance, if a single slab is encoded with 10 shots and an acceleration factor of *R*_*y*_ = 3 is applied along the phase-encoding direction, the under-sampling factor for each shot would amount to 30. Furthermore, the construction of Hankel matrices using 3D volumes in Eq. 7 would entail substantial computational costs and feasibility concerns.

### C Self-navigated 3D multi-slab imaging framework

Our method, illustrated in Fig. 1b, aims to integrate the SLR reconstruction into 3D multi-slab diffusion imaging to eliminate the need for navigator acquisition. This is achieved with an extended CAIPI [17] sampling trajectory where each shot covers a wide range of kz planes and intersects with the central kz=0 plane (the intersections are called “self-navigation points” hereafter). The self-navigation points are used to reconstruct a fully sampled kz=0 plane for each shot, akin to a 2D navigator but without requiring additional scan time. The sampling trajectories of all shots are jointly optimized for robust self-navigation and multi-shot reconstruction. The limited self-navigation points result in high under-sampling. We developed a SPIRiT-based [18] SLR reconstruction that exploits the redundancy between coils and shots to produce a robust estimation of motion-induced phase error for each shot. This is then incorporated into the multi-shot reconstruction to eliminate shot-to-shot phase inconsistencies as in conventional 3D multi-slab diffusion imaging reconstruction [3–7] (Fig. 1).

### D K-space sampling optimization

**Fig 2:**
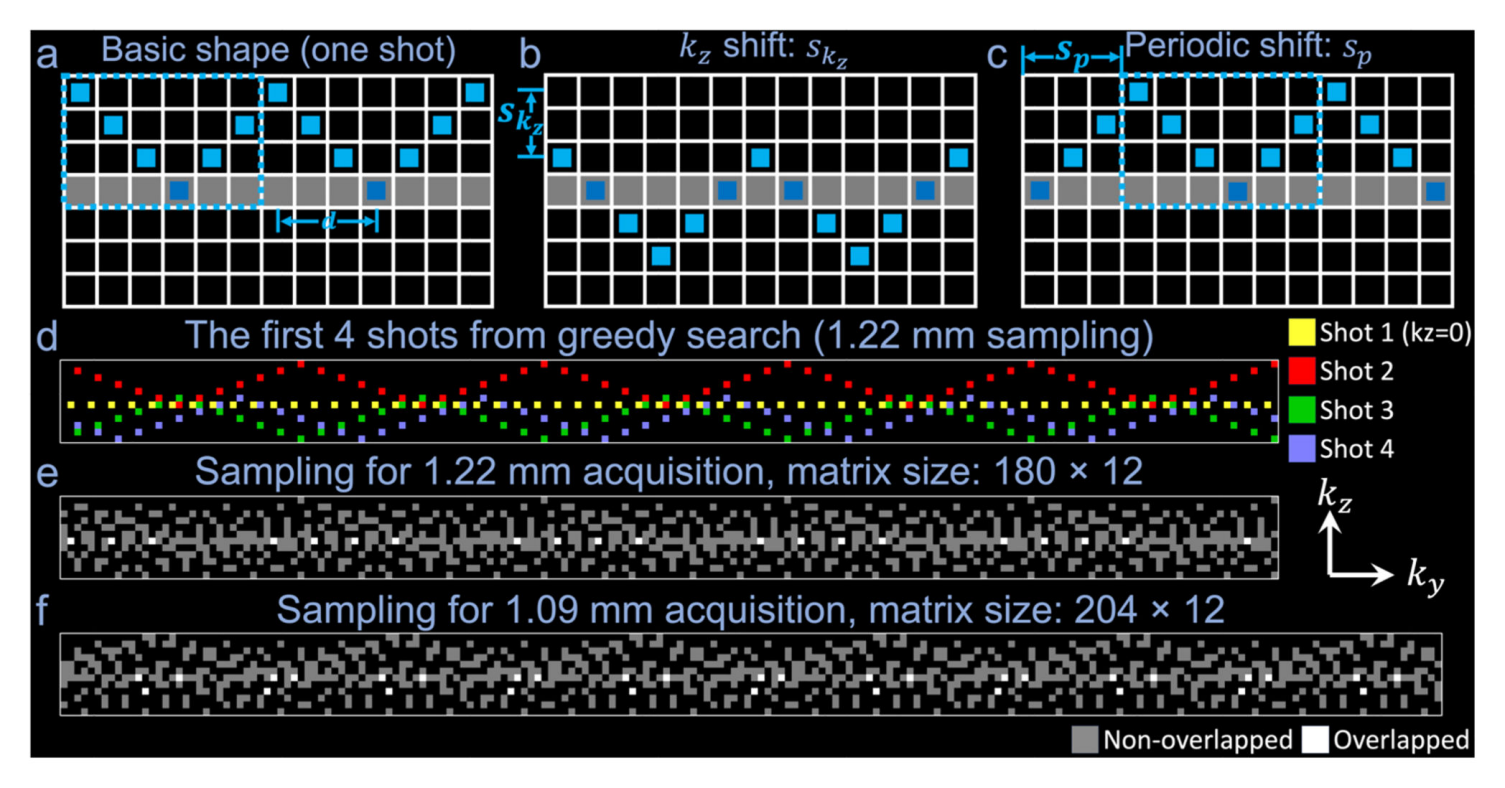
K-space sampling optimization. The basic shape of one shot of the proposed extended CAIPI sampling (a), the illustration of sampling parameters: kz shift $s_{k_{z}}$ (b) and periodic shift *s*_*p*_ (c). Note the parallel imaging acceleration along the phase encoding direction (ky) is not illustrated in (a-c) for simplicity, and acceleration factor=3 is used in (d-f). The intersections with central kz=0 plane (self-navigation points) are marked in dark blue (a-c). The parameter *d* which denotes the shortest distance between self-navigation points and the ky-kz center is also illustrated in (a). The dashed boxes indicate one period of the periodic sampling (a, c). The first 4 shots of resulting sampling patterns for 1.22 mm acquisition are plotted in yellow (kz=0 traversal shot), red, green, and blue, respectively (d). The overall sampling for 1.22 mm (matrix size: 180×12, e) and 1.09 mm acquisition (matrix size: 204×12, f) from the proposed sampling optimization are demonstrated. The non-overlapped and overlapped points are marked in gray and white, respectively.

The sampling coverage of each shot’s trajectory is expanded along kz by inserting kz blips between readout gradients, such that each shot traverses through the central kz=0 plane to provide the self-navigation points. The kz width of each shot *w* was set to $w=\text{floor}\left(N_{k_{z}}/2\right)+1$, the minimal value to ensure each shot intersects with the central kz=0 plane. Using this value can minimize the period of the sampling and therefore maximize the number of self-navigation points to benefit the 2D phase map reconstruction. A fundamental sampling configuration was established for an individual shot with a fixed ky period and kz width (Fig. 2a), based on which the sampling pattern was designed.

Drawing from this basic sampling, each shot could be acquired with a different pattern by modifying several parameters characterizing the sampling. The kz shift $s_{k_{z}}$ can be altered to determine the sampling’s vertical position (Fig. 2b) $\left(s_{k_{z}}\in \left[0,N_{k_{z}}-w\right]\right)$. The periodic shift *s*_*p*_ controls the starting point of the periodic sampling (Fig. 2c). With a kz width of *w*, the period of the sampling would be 2*w* − 2. Therefore, the range of *s*_*p*_ is [0, 2*w* − 3]. Parallel imaging acceleration is usually applied along the phase encoding direction (ky) to shorten TE and mitigate geometric distortion (not illustrated in Fig. 1 and Fig. 2a, b, c). Therefore, the sampling pattern can also be modified with a ky shift $s_{k_{y}}(s_{k_{y}}\in \left[0,1,\ldots ,R_{y}-1\right]$, where *R*_*y*_ is the parallel imaging acceleration factor along ky).

The sampling pattern can impact the image reconstruction quality in various ways. First, distinct shots might overlap, reducing sampling efficiency. Second, large gaps in k-space coverage could introduce substantial artifacts in the reconstructed images [19]. Lastly, the positioning of self-navigation points also affects phase correction performance. Ideally, each shot should incorporate some self-navigation points positioned close to the ky-kz center to capture sufficient low-frequency data for precise 2D phase map reconstruction. An optimal sampling scheme should strive to minimize overlap and gaps in k-space coverage, while ensuring each shot includes some self-navigation points near the ky-kz center.

We developed a framework to optimize the sampling pattern through metrics of overlap, gaps, and self-navigation points. The metric *o*_*i*_ is the number of overlap points in the *i*-shot-combined sampling, where *i* is the optimization step count and *i* ∈ [1, *N*_*shot*_]:

(9) $$o_{i}=T_{o}\left(\overset{i}{\underset{j=1}{\sum }}M_{j}(s_{k_{y_{j}}},s_{k_{zj}},s_{p_{j}})\right),$$

where $M_{j}(s_{k_{y_{j}}},s_{k_{zj}},s_{p_{j}})$ is the sampling mask for shot *j*, specified by parameters $s_{k_{y_{j}}},s_{k_{zj}},s_{p_{j}}$, respectively. *T*_*o*_ denotes an operation counting the number of elements greater than one within the combined sampling mask $\Sigma_{j=1}^{i}M_{j}$.

The metric *g*_*i*_ reflects the number of gaps larger than 3×3 in the *i*-shot-combined sampling:

(10) $$g_{i}=T_{g}\left(\left(\sum_{j=1}^{i}M_{j}\left(s_{k_{y_{j}}},s_{k_{zj}},s_{p_{j}}\right)\right)*\left[\begin{array}{ccc} 1 & 1 & 1\\ 1 & 1 & 1\\ 1 & 1 & 1 \end{array}\right]\right),$$

where *T*_*g*_ counts the number of zeros in the convolution result.

The metric *d* is the shortest distance between self-navigation points and the ky-kz center for each shot (Fig. 2a). It is essential for each shot to have a small value of *d* to provide low-frequency information for accurate reconstruction of 2D phase maps. We establish a maximum allowable distance *d*_max_.

We optimize the sampling by solving the following problem:

(11) $$\text{arg}\underset{s_{k_{y_{i}}{,}^{s}k_{z_{i}}{,}^{s}p_{i}}}{\text{min}}o_{i}+g_{i}+d_{i},\;s.t.,\;d_{i}\leq d_{\text{max}}.\;$$

The optimization is solved efficiently using greedy search in a shot-by-shot manner. Importantly, the first shot is designed to traverse the kz=0 plane without kz blip to accurately capture the magnitude information of kz=0, which benefits the SLR reconstruction of the phase maps (see Section E).

### E SPIRiT-based SLR reconstruction for phase map estimation

We address the extreme under-sampling of the phase maps by jointly leveraging the shared information across shots and coils using a SPIRiT [18, 20]-based SLR reconstruction.

**Fig. 3:**
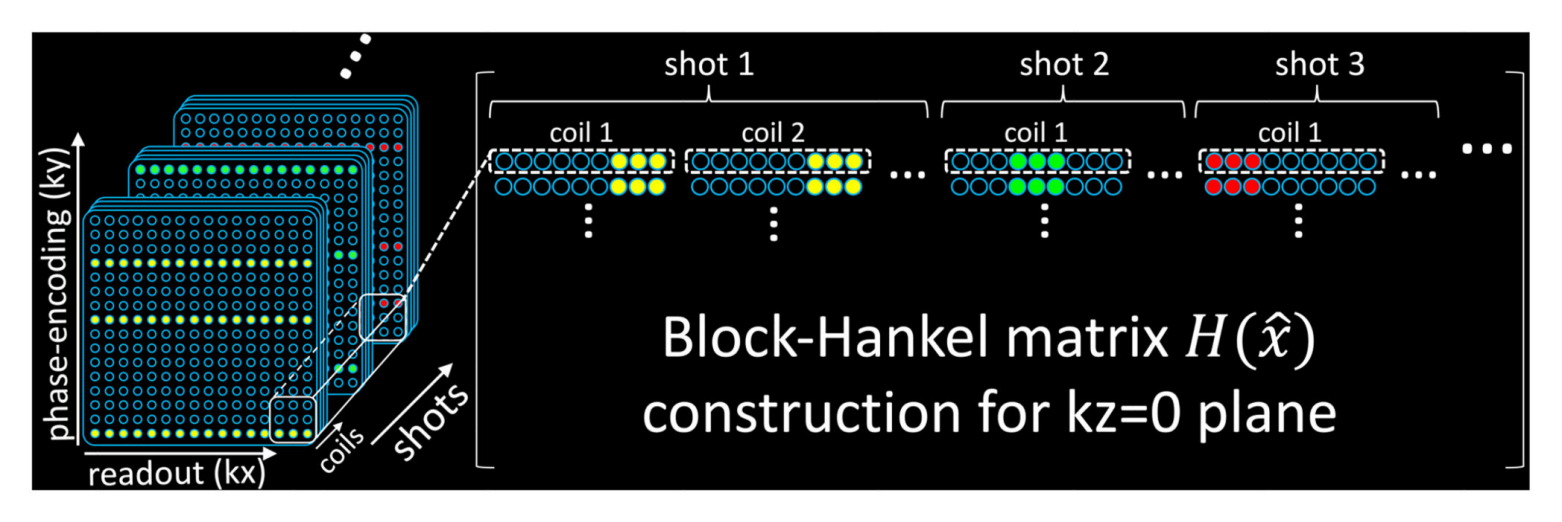
The block-Hankel matrix for the kz=0 plane reconstruction leverages the low-rank properties in both coil and shot dimensions. The sampled lines represent the self-navigation points in the kz=0 plane, reflecting the under-sampling without the parallel imaging acceleration. The actual sampling includes an additional parallel imaging acceleration factor=3, resulting in a threefold increase in the overall under-sampling factor.

The careful design of the sampling facilitates the adaptation of 2D SLR method for the reconstruction of the central kz=0 plane for each shot. Equations 3-8 can be readily applied to construct a low-rank block-Hankel matrix for kz=0 data, assuming *x*_*j*_ represents the kz=0 image for shot *j*. Notably, unlike the original 2D SLR formulation [11, 12, 14] where *x*_*j*_ is the coil-combined image, we utilize the multi-coil data to construct the block-Hankel matrix. Because coil sensitivity is smooth with limited k-space support, the multi-coil formulation promotes the low-rankness of the block-Hankel matrices, and therefore is believed to be beneficial for the parallel imaging reconstruction [21]. The multi-shot, multi-coil block-Hankel matrix of the kz=0 plane $H(\hat{x})$ is formulated as (Fig. 3):

(12) $$H(\hat{x})=\left[\begin{array}{c} H_{shot}({\hat{x}}_{1})\;H_{shot}\left({\hat{x}}_{2}\right)\ldots H_{shot\;}\left({\hat{x}}_{N_{shot}}\right) \end{array}\right]$$

(13) $$H_{shot}\left({\hat{x}}_{u}\right)=\left[\begin{array}{c} H_{1}({\hat{x}}_{u,1})\;H_{1}\left({\hat{x}}_{u,2}\right)\;\ldots \;H_{1}\left({\hat{x}}_{u,N_{coil}}\right) \end{array}\right]$$

where $H_{shot\;}\left({\hat{x}}_{u}\right)$ is the multi-coil submatrix for shot *u*, ${\hat{x}}_{u,v}$ is the k-space data from the *u*^*th*^ shot and v^*th*^ coil, and *N*_*coil*_ denotes the total number of coils. The limited k-space support of the coil sensitivity resulting from its smoothness causes $H_{shot\;}\left({\hat{x}}_{u}\right)$ to be rank-deficient [21], with $\text{rank}\left(H_{shot\;}\left({\hat{x}}_{u}\right)\right)\leq {\left(w_{H}+s-1\right)}^{2}$ where *w*_*H*_ is the kernel size for constructing the block-Hankel matrix, and *s* is the k-space support size of the coil sensitivity. This induces coil low-rankness in addition to shot low-rankness demonstrated by the annihilation relations in Eqs. 3-8. By minimizing $||H(\hat{x})|{|}_{*}$, we enforce low-rank and harness the redundancy in both the shot and coil dimensions. This constraint promotes similarity among the magnitude images derived from different shots. The use of one shot that traverses the kz=0 plane without kz blips provides accurate magnitude information m’ to improve the robustness and accelerate the convergence.

Our SPIRiT-based SLR reconstruction recovers the 2D phase maps for each shot from the self-navigation points by solving:

(14) $$\text{arg}\;\underset{\hat{x}}{\text{min}}{\left|\left|{ℳ}_{k_{z}0}\hat{x}-y_{k_{z}0}\right|\right|}_{2}^{2}+\lambda_{1}{\left|\left|\left(G_{k_{z}0}-I\right)\hat{x}\right|\right|}_{2}^{2}+\lambda_{2}{\left|\left|H\left(\hat{x}\right)\right|\right|}_{*},$$

where ${ℳ}_{k_{z}0}$ selects self-navigation points, $\hat{x}$ is the fully-sampled multi-coil k-space data for all shots at kz=0, $y_{k_{z}0}$ is the acquired self-navigation points, $G_{k_{z}0}$ is the SPIRiT kernel trained on the kz=0 calibration data (see below), *I* is the identity matrix, and *λ*_1_, *λ*_2_ are the hyperparameters for the SPIRiT and SLR constraints.

The SPIRiT kernel $G_{k_{z}0}$ is trained on calibration data similarly to GRAPPA [22] and performs convolutions in k-space to reach self-consistency:

(15) $$\hat{x}=G_{k_{z}0}\hat{x},$$

which is achieved by minimizing $||\left(G_{k_{z}0}-I\right)\hat{x}|{|}_{2}^{2}$ in Eq. 14.

We reformulate Eq. 14 to solve it iteratively using alternating direction method of multipliers (ADMM) [23]:

(16) $$\begin{array}{c} \text{arg}\;\underset{\hat{x}}{\text{min}}{\left|\left|{ℳ}_{k_{z}0}\hat{x}-y_{0}\right|\right|}_{2}^{2}+\lambda_{1}{\left|\left|\left(G_{k_{z}0}-I\right)\hat{x}\right|\right|}_{2}^{2}+\lambda_{2}{\left|\left|Z\right|\right|}_{*},\\ s.t.,\;Z-H\left(\hat{x}\right)=0. \end{array}$$

In the *k*^*th*^ iteration, Eq. 16 is further split into the following three subproblems:

(17) $$\begin{array}{c} {\hat{x}}^{k}=\arg\;\underset{\hat{x}}{\min}{\left|\left|{ℳ}_{k_{z}0}\hat{x}-y_{k_{z}0}\right|\right|}_{2}^{2}+\lambda_{1}{\left|\left|\left(G_{k_{z}0}-I\right)\hat{x}\right|\right|}_{2}^{2}+\\ \left(\frac{\beta }{2}\right){\left|\left|z^{k-1}-H\left(\hat{x}\right)+u^{k-1}\right|\right|}_{2}^{2}, \end{array}$$

(18) $$z^{k}=\text{arg}\;\underset{\text{z}}{\text{min}}\;\lambda_{2}{\left|\left|z\right|\right|}_{*}+\left(\frac{\beta }{2}\right){\left|\left|z-H\left({\hat{x}}^{k}\right)+u^{k-1}\right|\right|}_{2}^{2},$$

(19) $$u^{k}=u^{k-1}+z^{k}-H\left({\hat{x}}^{k}\right),$$

where *β* is the dual update step length. As all shots share the same magnitude information, an explicit magnitude image m’ for kz=0 is obtained from the kz=0 traversing shot, which is included in Eq. 17 as another constraint:

(20) $$\begin{array}{c} {\hat{x}}^{k}=\text{arg}\;\underset{\hat{x}}{\text{min}}\;{\left|\left|{ℳ}_{k_{z}0}\hat{x}-y_{0}\right|\right|}_{2}^{2}+\lambda_{1}{\left|\left|\left(G_{k_{z}0}-I\right)\hat{x}\right|\right|}_{2}^{2}+\\ \lambda_{3}{\left|\left|\hat{x}-F{\text{m}}^{\prime }\Phi^{k-1}\right|\right|}_{2}^{2}+\left(\frac{\beta }{2}\right){\left|\left|z^{k-1}-H\left(\hat{x}\right)+u^{k-1}\right|\right|}_{2}^{2}, \end{array}$$

where Φ^*k*–1^ is the image phase of ${\hat{x}}^{k-1}$. In practice, the constraint $||\hat{x}-F{\text{m}}^{\prime }\Phi^{k-1}|{|}_{2}^{2}$ was found effective in improving the robustness and convergence speed of the reconstruction. We solve Eq. 18 by applying singular value decomposition (SVD) and hard thresholding similar to previous work [11, 21, 24]. Eq. 20 is solved using the conjugate gradient (CG) method.

A 2D phase map can then be extracted from the reconstructed kz=0 image ${\hat{x}}_{j}$ for each shot:

(21) $$\phi_{d_{j}}=\frac{S^{H}F^{-1}{\hat{x}}_{j}}{\left||S^{H}F^{-1}{\hat{x}}_{j}|\right|},j\in \left[1,N_{shot}\right],$$

where *S* is the coil sensitivity maps for kz=0, which can be obtained from kz=0 calibration data. According to Eq. 2, the 2D phase maps can be incorporated into the forward model to address the phase variations in the multi-shot reconstruction:

(22) $$\hat{X}=\text{arg}\;\underset{\hat{X}}{\text{min}}\;\sum_{j}{\left|\left|{ℳ}_{j}F\phi_{d_{j}}F^{-1}\hat{X}-y_{j}\right|\right|}_{2}^{2}+\lambda_{4}{\left|\left|\left(G_{slab}-I\right)\hat{X}\right|\right|}_{2}^{2},$$

where $\hat{X}$ is the phase error-corrected 3D k-space, *G*_*slab*_ is the SPIRiT kernel trained on coil calibration data for the whole slab, and *λ*_4_ is the SPIRiT regularization weight of the final phase-corrected reconstruction. The desired slab image can be obtained from the sum-of-squares of $F^{-1}\hat{X}$.

## III Methods

### A Data acquisition

A 3D multi-slab spin-echo diffusion MRI sequence [4] was modified to integrate the proposed CAIPI sampling for self-navigation (referred to as “Self-nav CAIPI” hereinafter).We used $N_{k_{z}}=12$, *R*_*y*_ =3, *d*_max_ =15, such that $s_{k_{z}}\in [0,5],s_{k_{y}}\in [0,2]$, *s*_*p*_ ∈ [0,11]. *N*_*shot*_ was also set to 12 for consistency with the conventional 3D multi-slab imaging. In the *i*^*th*^ step of the sampling optimization, we exhaustively explored all combinations of $s_{k_{y_{i}}},s_{k_{z_{i}}},s_{p_{i}}$ to identify lowest value for the cost function *o*_*i*_ + *g*_*i*_ + *d*_*i*_. The resulting sampling patterns for 1.22 mm acquisition (matrix size: 180×12 for ky-kz plane) and 1.09 mm acquisition (matrix size: 204×12 for ky-kz plane) are demonstrated in Fig. 2d, e and Fig. 2f. There is little overlapping and no substantial k-space gaps. The samplings are denser near k-space center with an overall random distribution.

Some experiments included an explicit navigator acquisition with 64 phase encoding lines and matching the imaging echo for phase encode direction, Ry, and echo spacing (ES).

Subjects were scanned on a Siemens 7T scanner (Siemens Magnetom, Erlangen, Germany) using a 32-channel receive coil. Written informed consent in accordance with local ethics was obtained from each subject.

Table I summarizes six experimental protocols. Images were acquired at 1.22 and 1.09 mm isotropic resolutions (180×180 and 204×204 matrix, ES=0.76 and 0.82 ms, respectively). Unless otherwise specified, acquisitions used 13 slabs, 12 slices/slab, 2 slice slab overlap, partial Fourier (PF) factor 3/4 and b=1000 s/mm^2^.

#### Experiment 1 Simulation Evaluation

To validate the proposed sampling and reconstruction, we acquired single-slab data (12 slices) from one subject at 1.22 mm (Table I. 1A) and 1.09 mm (Table I. 1B). Fully sampled reference data with b=1000 s/mm^2^ were reconstructed from three scans (each with *R*_*y*_ =3 and the conventional sampling, phase error-corrected using the 2D navigator) with 0, 1, and 2Δ*k*_*y*_ shift. Realistic phase-corrupted multi-shot data were simulated by multiplying the reference data with one set of navigator phase maps followed by applying the shot sampling masks.

Using the fully sampled dataset, we conducted the following experiments to evaluate our sampling and reconstruction:

- Exp 1.1: we evaluated conventional and Self-nav CAIPI sampling (Fig. 2e, f).
- Exp 1.2: we simulated three sub-optimal samplings by solving Eq. 11 (i) without the overlap constraint *o*_*i*_ (i.e., *g*_*i*_ + *d*_*i*_ is minimized subject to *d*_*i*_ ≤ *d*_max_); (ii) without the k-space gap constraint *g*_*i*_ (i.e., *o*_*i*_ + *d*_*i*_ is minimized subject to *d*_*i*_ ≤ *d*_max_); (iii) without the self-navigation performance constraint *d*_*i*_ (i.e., *o*_*i*_ + *g*_*i*_ is minimized), and evaluated the reconstructed images.
- Exp 1.3: we simulated Self-nav CAIPI sampling with and without the use of a non-blipped kz=0 shot and investigated the impact of including the magnitude constraint $||\hat{x}-F{\text{m}}^{\prime }\Phi^{k-1}|{|}_{2}^{2}$ in Eq. 20.
- Exp 1.4: we compared our method with a previously proposed self-navigation method by Moeller et al. [9] (entitled “Self-nav Conventional”).
- Exp 1.5: we investigated the contribution of shot and coil dimension in our SLR reconstruction and implemented SLR only leveraging redundancy across shots (i.e., coil-combined SLR) or coils; we also selected 16-coil data from our 32-coil data to evaluate the performance of our method when fewer coils are available.

#### Experiment 2 SNR Evaluation with Matched TR

To quantitatively evaluate SNR, we acquired data from three subjects using conventional (Table I. 2A) and Self-nav CAIPI sampling (Table I. 2B) with matched TE and TR. To evaluate the accuracy of phase map estimation, navigators were also acquired. Diffusion weighting along the readout, phase, and slice directions was evaluated, each with twelve repetitions.

#### Experiment 3 SNR Efficiency Evaluation with Optimal TR

To quantitatively compare the SNR efficiency, we acquired data in four subjects with conventional (with navigators, Table I. 3A) and Self-nav CAIPI (without navigators, Table I. 3B) sampling, with different TR optimized for each scan. Diffusion weighting was along readout with twelve repetitions.

#### Experiment 4 DTI Comparison

To compare DTI results, we acquired data in two subjects with 16 diffusion encoding directions with the conventional sampling (with navigators, Table I. 4A) and the Self-nav CAIPI sampling (without navigators, Table I. 4B). The two b=0 images were acquired along opposite phase encoding directions (1 blip-up and 1 blip-down) using conventional rectangular sampling.

#### Experiment 5 High-b-value Protocol

To demonstrate higher b-values, we acquired data from one subject with the conventional sampling (Table I. 5A, C) and the Self-nav CAIPI sampling (Table I. 5B, D) (both with navigators) at b=2000 s/mm^2^ (Table I. 5A, B) and b=3000 s/mm^2^ (Table I. 5C, D).

#### Experiment 6 Tractography Protocol

To demonstrate tractography analysis, we acquired data in one subject with Self-nav CAIPI sampling and 48 diffusion directions along with 6 b=0 images (3 blip-up and 3 blip-down) (Table I. 6).

### B Reconstruction details

The image reconstruction was conducted in MATLAB 2021a (Mathworks, Natick, MA, USA). The SPIRiT kernels $G_{k_{z}0}$ and $G_{k_{z}0}$ were trained using a whole-brain gradient echo coil calibration scan (~1 min acquisition time). To train $G_{k_{z}0}$, the calibration data for each slab were Fourier transformed along the slice direction, and the central kz=0 plane data were used. The kz=0 coil sensitivity in Eq. 21 was estimated from the same data employed to train $G_{k_{z}0}$ using ESPIRiT [25]. To obtain *G*_*slab*_, the same calibration data was Fourier transformed along kx, and *G*_*slab*_ was trained for each ky-kz plane. All 32-coil k-space data were compressed to 8 coils [26].

For the 3D data reconstruction in Eq. 22, the k-space data were first Fourier transformed along kx followed by reconstruction performed for each ky-kz plane using the SPIRiT kernel *G*_*slab*_. The reconstructed 2D images were concatenated along the readout direction to form the whole image volume. The kernel sizes for $G_{k_{z}0}$ and *G*_*slab*_ were both set to 5×5. The kernel size *w*_*H*_ for constructing $H(\hat{x})$ was set to 10 (i.e., 10×10 kernel). The hyperparameters *λ*_1_, *λ*_2_, *λ*_4_, *β* were empirically selected as 1, 1e-4, 1e-4, 10, respectively. The SVD thresholding for solving Eq. 18 was performed by retaining the largest N singular values and their corresponding vectors, where $N=w_{H}^{2}$ (i.e., N=100 in this study). The ADMM iteration number was set to 50. The CG iteration numbers for solving Eqs. 20 and 22 were both set to 30.

The 2D navigator images were reconstructed with 2D GRAPPA. Because the phase variations are expected to be spatially smooth [10], the navigator images were filtered by a k-space Hamming window of size 32×32 to reduce noise. The phase images were extracted as an estimation of motion induced phase errors. The SLR estimated *ϕ*_*d*_ were also filtered using the same Hamming window for the reconstruction in Eq. 22.

In Exp 1.4, the implementation of “Self-nav Conventional” followed the steps detailed in [9], except a stronger Gaussian filter with *σ* = 20 was used in the final step to eliminate the high-frequency noise in the estimated phase map.

In Exp 1.5, for the coil-combined SLR, the multi-coil k-space data were combined using the kz=0 coil sensitivity map. The forward model, cost function, and construction of $H(\hat{x})$ followed the original 2D SLR configuration [11]. For the SLR only leveraging coil redundancy, the construction of $H(\hat{x})$ and the optimization of Eq. 14 were conducted in a shot-by-shot manner. Both methods used the ADMM optimization with the same parameters as mentioned previously. The 16-coil data were selected from the original 32-coil data using a greedy search, with the aim of finding a combination of 16 coils that closely matched the combined profile of all 32. At each step of the search, the mean absolute error between the sum-of-squares of the sensitivity maps from the selected coils and those from the full 32 coils was minimized.

The optimized sampling patterns and the codes for sampling optimizations and reconstructions are openly available at: https://github.com/liziyu0929/Self-nav_CAIPI.

### C Image analysis

Image post-processing was conducted using the FMRIB Software Library (FSL) [27] unless indicated otherwise. For Exp. 1, the reconstructed images from different methods were evaluated by calculating normalized root mean squared error (NRMSE) with the fully sampled ground truth data. For Exp. 2, 3, and 5, the whole-brain diffusion-weighed data were obtained by directly combining multiple slabs and averaging the overlapped slices. The SNR and SNR efficiency were calculated using the multi-repetition DWIs acquired in Exps. 2 and 3. To mitigate the impact of subject motion on SNR evaluation, all multi-repetition data were co-registered to the first repetition using “flirt” [28] with 6 degrees of freedom and trilinear interpolation. An interim mean image was computed by averaging all co-registered data, and the two volumes with the highest NRMSE with respect to this interim mean image were discarded to reduce motion contamination. The remaining ten-repetition data, denoted as *S*_10_, were used for SNR calculation. The voxel-wise SNR map was calculated as mean(*S*_10_)./std(*S*_10_) (./ denotes the element-wise division). The SNR efficiency (i.e., SNR per unit time) was calculated as $SNR/\sqrt{TR}$. The mean SNR and SNR efficiency were then calculated by taking the mean of the SNR and SNR efficiency maps within a brain mask, respectively.

For DTI and tractography experiments (Exp. 4 and 6), slab combination and correction for slab saturation artifacts were performed for the diffusion data using nonlinear inversion of slab profile encoding (NPEN) [29]. Whole-brain images were corrected for Gibbs ringing [30]. A whole-brain field map was estimated using blip-reversed b=0 image volumes using “topup” [31], which was then input to “eddy” [32] along with all diffusion data to correct for off-resonance distortions, eddy current effects, and subject motion. The diffusion analyses were conducted in the native diffusion space. For Exp. 4, the diffusion tensor model fitting was performed using “dtifit”. For Exp. 6, white matter tractography was performed using “autoPtx” [33] including probabilistic model fit “bedpostx” and probabilistic tractography “probtrackx”.

## IV Results

**Fig. 4:**
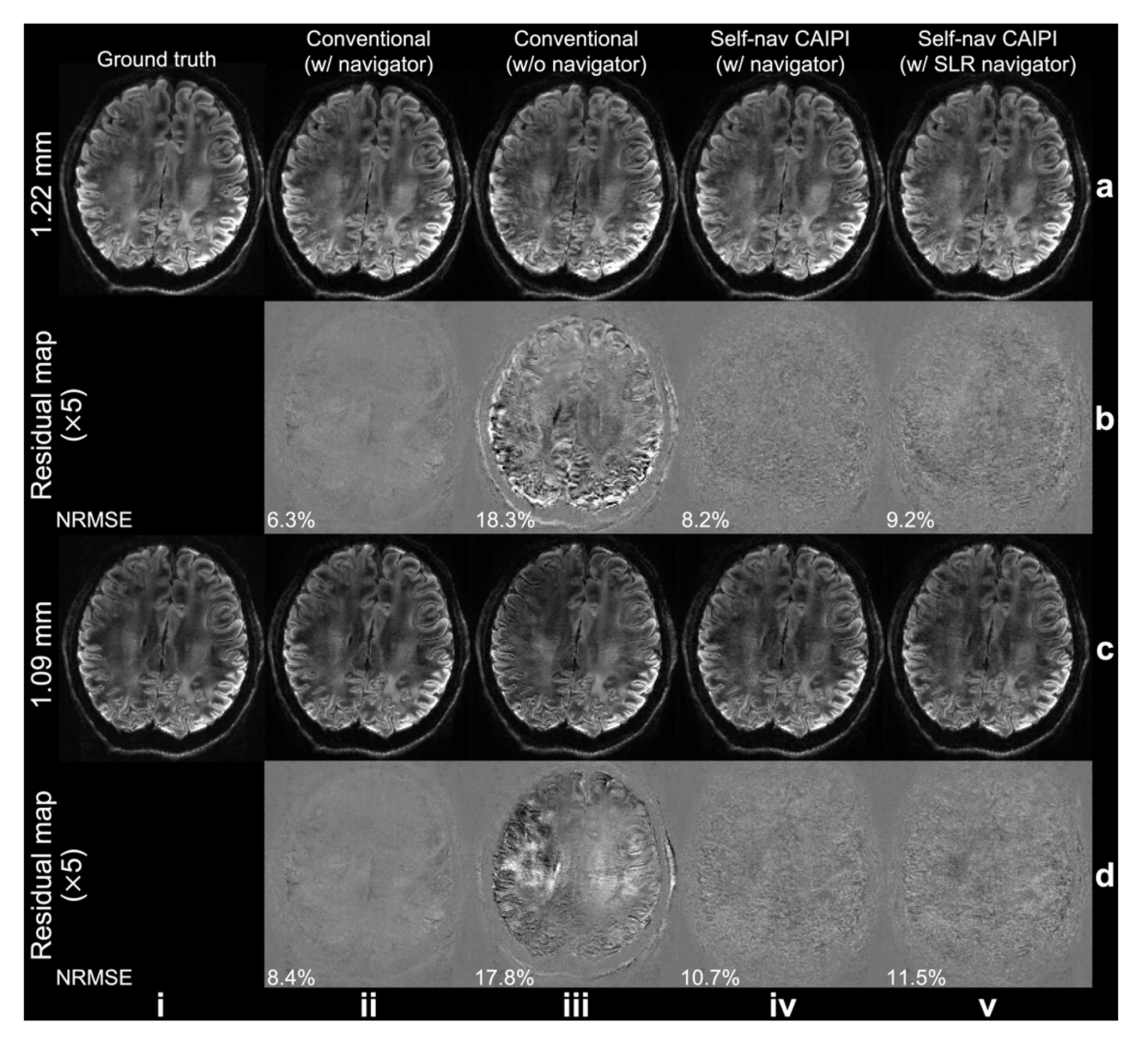
Image reconstruction results from the simulation experiment. Retrospectively under-sampled images from Simulation Evaluation (Exp. 1.1, slab-center slices) are shown, reconstructed using: conventional sampling with ground truth phase maps (ii), conventional sampling without phase error correction (iii), self-navigated CAIPI sampling with ground truth phase maps (iv), and self-navigated CAIPI sampling with structured low-rank estimated phase maps (v), and at 1.22 mm (a) and 1.09 mm (c) isotropic resolutions, with residual maps (b and d) showing difference with the ground truth. The normalized root mean squared errors (NRMSE) of the entire slab are listed.

Fig. 4 demonstrates the image reconstruction results from the simulation data. For conventional sampling, the reconstructed images without phase error correction suffer from severe artifacts and exhibit high NRMSE (Fig. 4, iii). Employing navigator phase maps leads to significantly better image quality but requires additional scan time (Fig. 4, ii). In contrast, our proposed self-navigated approach avoids the necessity for navigators while achieving comparable efficacy in phase error correction with conventional navigated acquisition (Fig. 4, v). The optimized Self-nav CAIPI sampling produces similar image quality with navigator phase (Fig. 4, iv) and SLR-estimated phase (Fig. 4, v), demonstrating the robust phase variance estimation of the self-navigated approach. The residual maps exhibit random-noise-like error (Fig. 4b, d, iv, v) without anatomical structure, likely due to the pseudo-random sampling patterns.

**Fig. 5:**
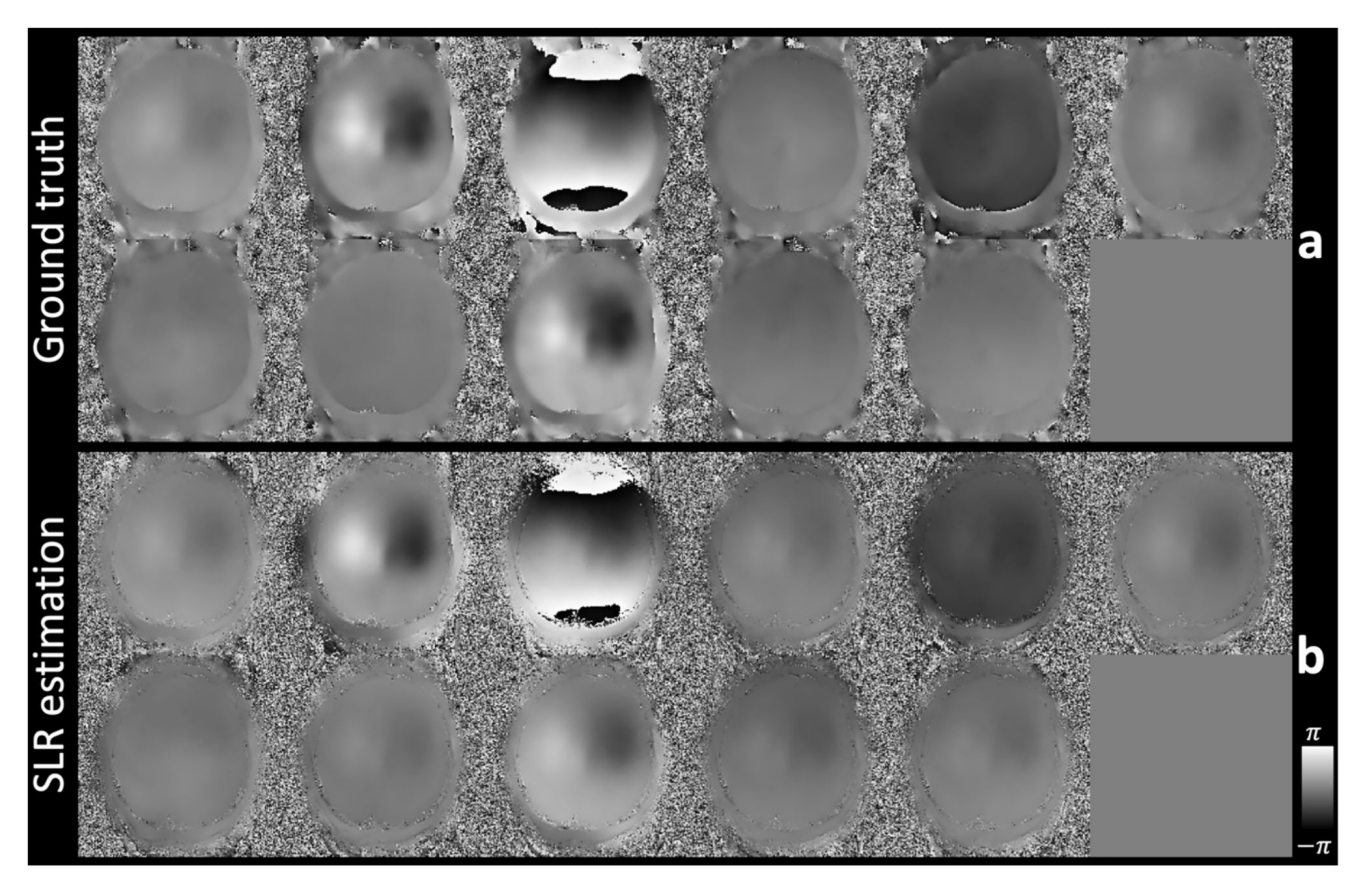
Phase map estimation from the simulation experiment. The ground truth phase maps (a) and structured low-rank (SLR) estimated phase maps (without filtering) from the proposed method (b) are shown for different shots of the simulated data from Simulation Evaluation (Exp. 1.1) at 1.22 mm isotropic resolutions. The phase of the shot traversing the entire kz=0 is subtracted from all shots to eliminate the phase offsets, effectively demonstrating the motion induced phase variations across shots (11 shots demonstrated).

The image reconstruction performance of the proposed method depends on the accuracy of the motion-induced phase variation maps. As show in Fig. 5, using the proposed SPIRiT SLR reconstruction, the estimated phase maps are highly similar to the ground truth phase maps with consistent spatial patterns for all shots, confirming the accuracy of our method’s motion induced phase error estimations.

**Fig. 6:**
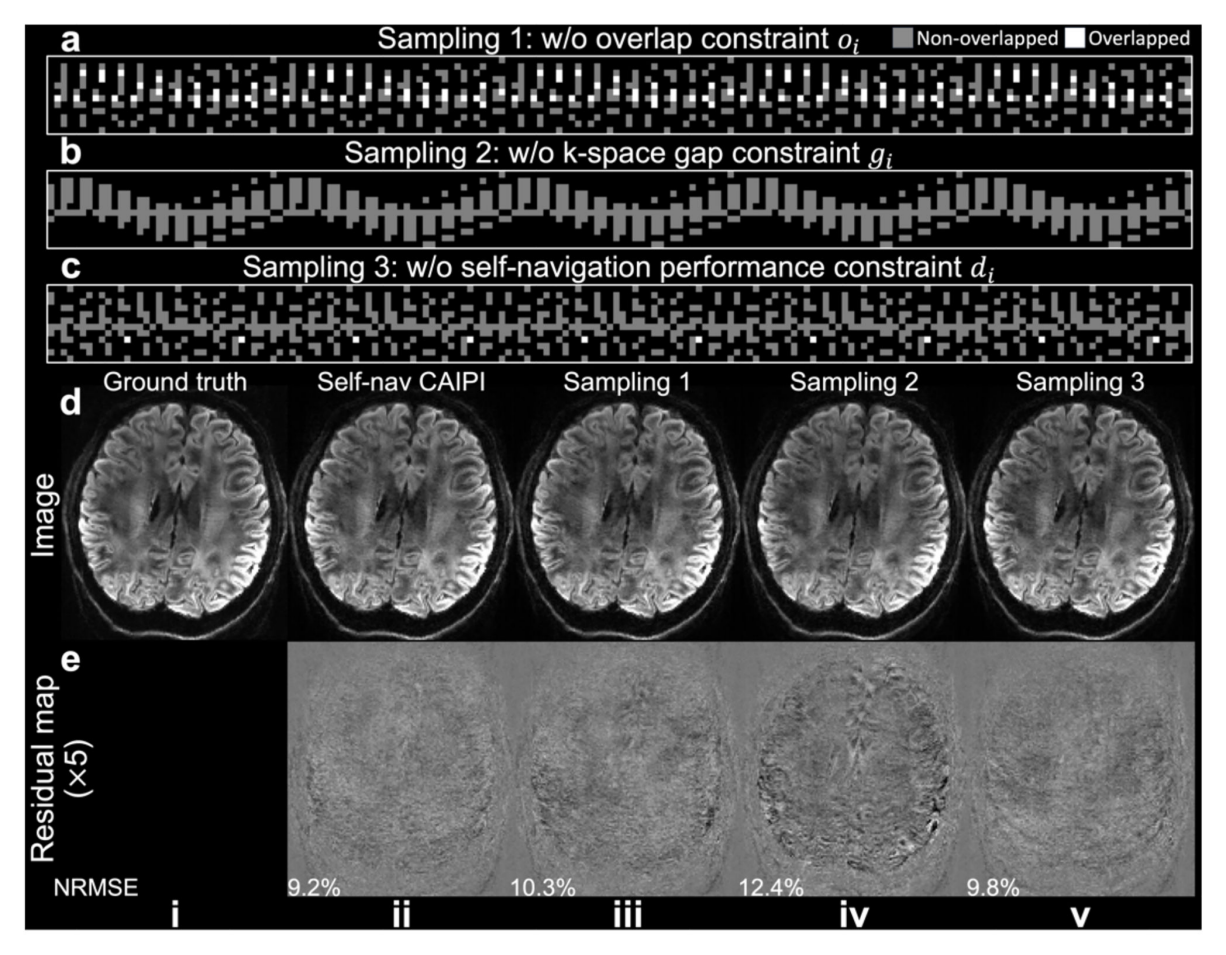
Evaluation of sampling optimization. Samplings for 1.22 mm acquisition are obtained by optimizing the cost function in Eq.11 without overlap constraint *o*_*i*_ (a), k-space gap constraint *g*_*i*_ (b), and self-navigation performance constraint *d*_*i*_ (c). Images (d) from Simulation Evaluation (Exp. 1.2, a slab-boundary slice) are under-sampled using the Self-nav CAIPI sampling (ii) and the three samplings in a-c (iii-v) and reconstructed with structured low-rank estimated phase maps, with residual maps (e) showing the difference with the ground truth.

Fig. 6 demonstrates the contribution of each constraint within our sampling optimization framework. The absence of the overlap constraint (*o*_*i*_) results in a sampling pattern with significant overlap (Fig. 6a), leading to higher NRMSE in the reconstructed images (Fig. 6d, e, iii). Omitting the k-space gap constraint (*g*_*i*_) produces sampling patterns with large gaps (Fig. 6b), exacerbating residual artifacts in the reconstructed images (Fig. 6d, e, iv). Removing the self-navigation performance constraint (*d*_*i*_) yields a uniform sampling pattern with little overlap (Fig. 6c). However, the reconstructed images exhibit slightly stronger aliasing and higher NRMSE (Fig. 6d, e, v) compared to Self-nav CAIPI, suggesting limitations in accurately estimating 2D phase maps due to insufficient low-frequency information contained in the self-navigation points of each shot.

**Fig. 7:**
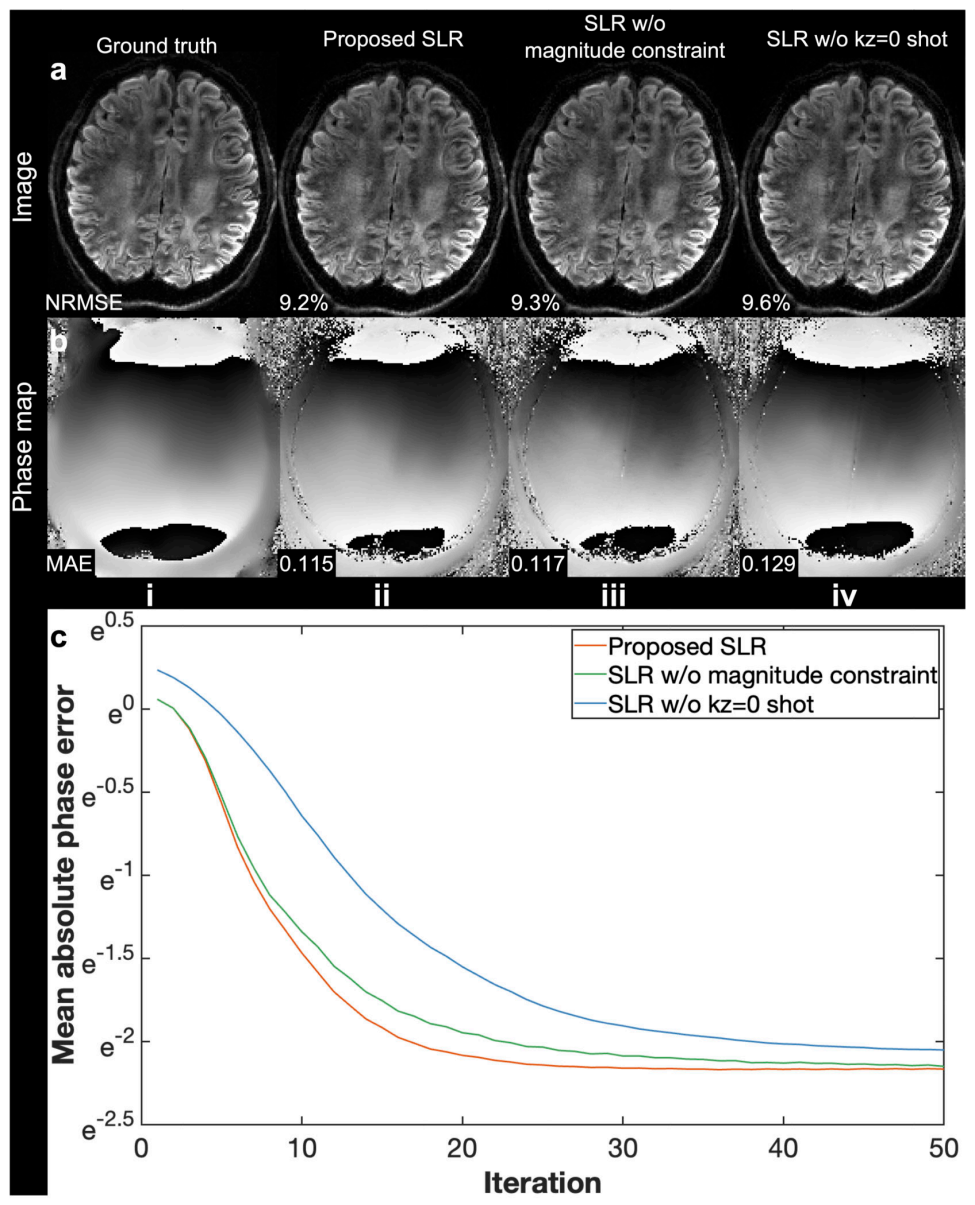
Impact of including the shot traversing the kz=0 plane. Retrospectively under-sampled images (a) and 2D phase maps (without filtering) (b) of one shot at 1.22 mm isotropic resolution from Simulation Evaluation (Exp. 1.3, a slab-center slice) are shown. Reconstructions using the proposed structured low-rank (SLR) (ii), SLR without the magnitude constraint $||\hat{x}-Fm^{\prime }\Phi^{k-1}|{|}_{2}^{2}$ in Eq. 20 but with kz=0 shot data (iii) and SLR without the kz=0 shot (substituted with a greedy-searched CAIPI shot) (iv) are compared. The mean absolute error (MAE) of phase maps of all shots within the brain mask are provided. The phase MAE plot of different SLR strategies at different ADMM iterations is also shown to demonstrate the convergence of the optimization (c).

Fig. 7 illustrates the impact of including the shot traversing the kz=0 plane. When the kz=0 traversing shot is replaced with another CAIPI shot obtained from greedy search in Eq. 11, the SLR reconstruction produces a less accurate phase map (Fig. 7b, iv) with a slower convergence (Fig. 7c). The lower phase accuracy affects the multi-shot reconstruction performance, leading to a higher reconstruction error (Fig. 7a, iv). Having the magnitude constraint $||\hat{x}-Fm^{\prime }\Phi^{k-1}|{|}_{2}^{2}$ in Eq. 20 is beneficial for further accelerating the convergence (Fig. 7c).

**Fig. 8:**
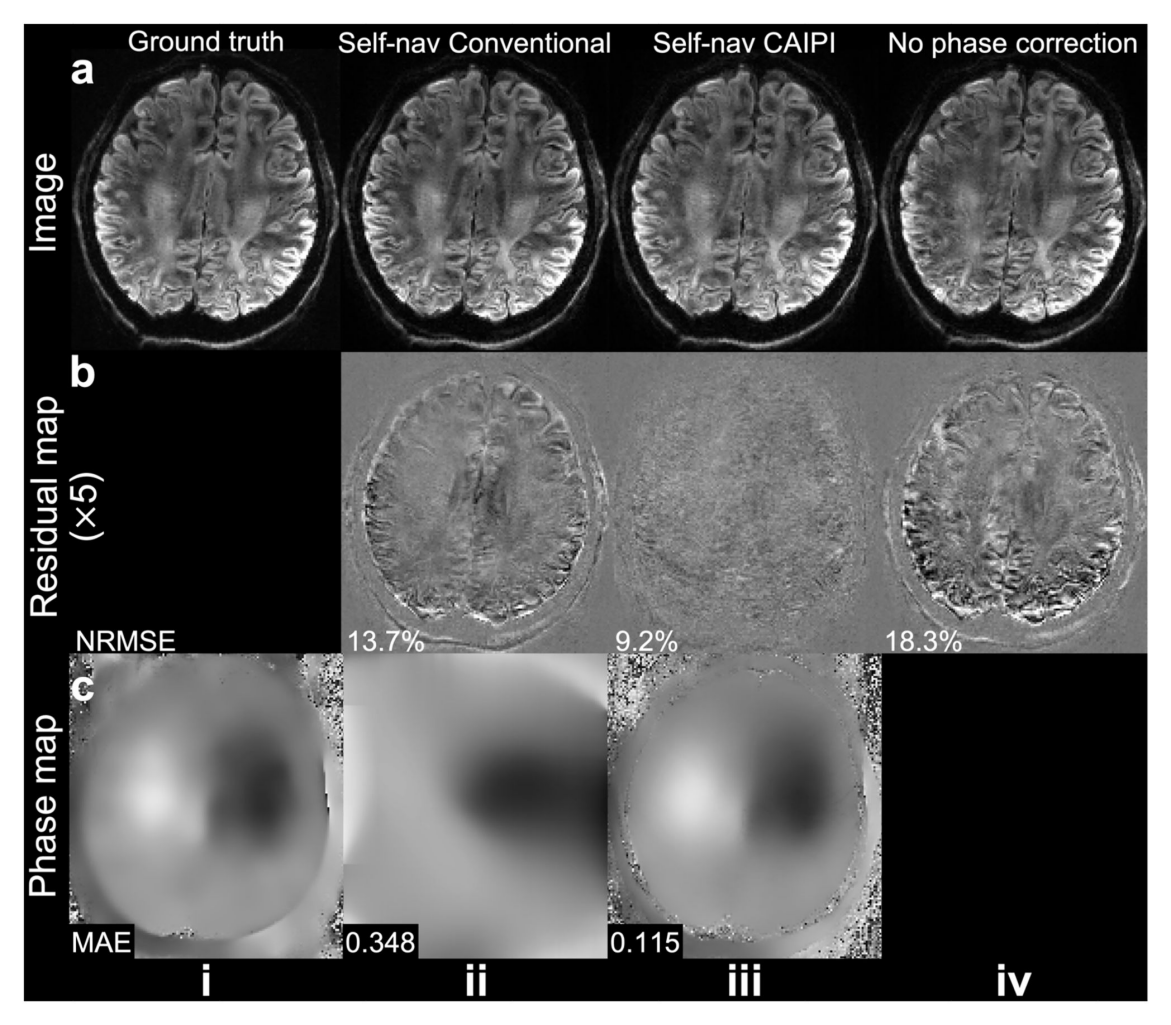
Comparison of self-navigation methods. Retrospectively under-sampled images (a), residual maps to ground truth (b), and estimated 2D phase maps of one shot (c) at 1.22 mm isotropic resolution from Simulation Evaluation (Exp. 1.4, a slab-center slice) from the method proposed by Moeller et al. [9] (entitled “Self-nav Conventional”, ii), our proposed method (Self-nav CAIPI, iii), and the conventional sampling without phase error correction (iv) are shown.

The proposed method achieves better self-navigation performance than the previous method proposed by Moeller et al. [9] (Fig. 8). Although their method provides improved image quality compared to the uncorrected image (Fig. 8, ii vs. Fig. 8, iv), its estimated phase map does not accurately capture the phase variation (Fig. 8c, ii), presumably due to the strong smoothing filter used to suppress the high-frequency noise from shots covering the peripheral k-space in conventional sampling. Self-nav CAIPI produces more accurate phase maps and image reconstruction with substantially lower NRMSE (Fig. 8, iii).

**Fig. 9:**
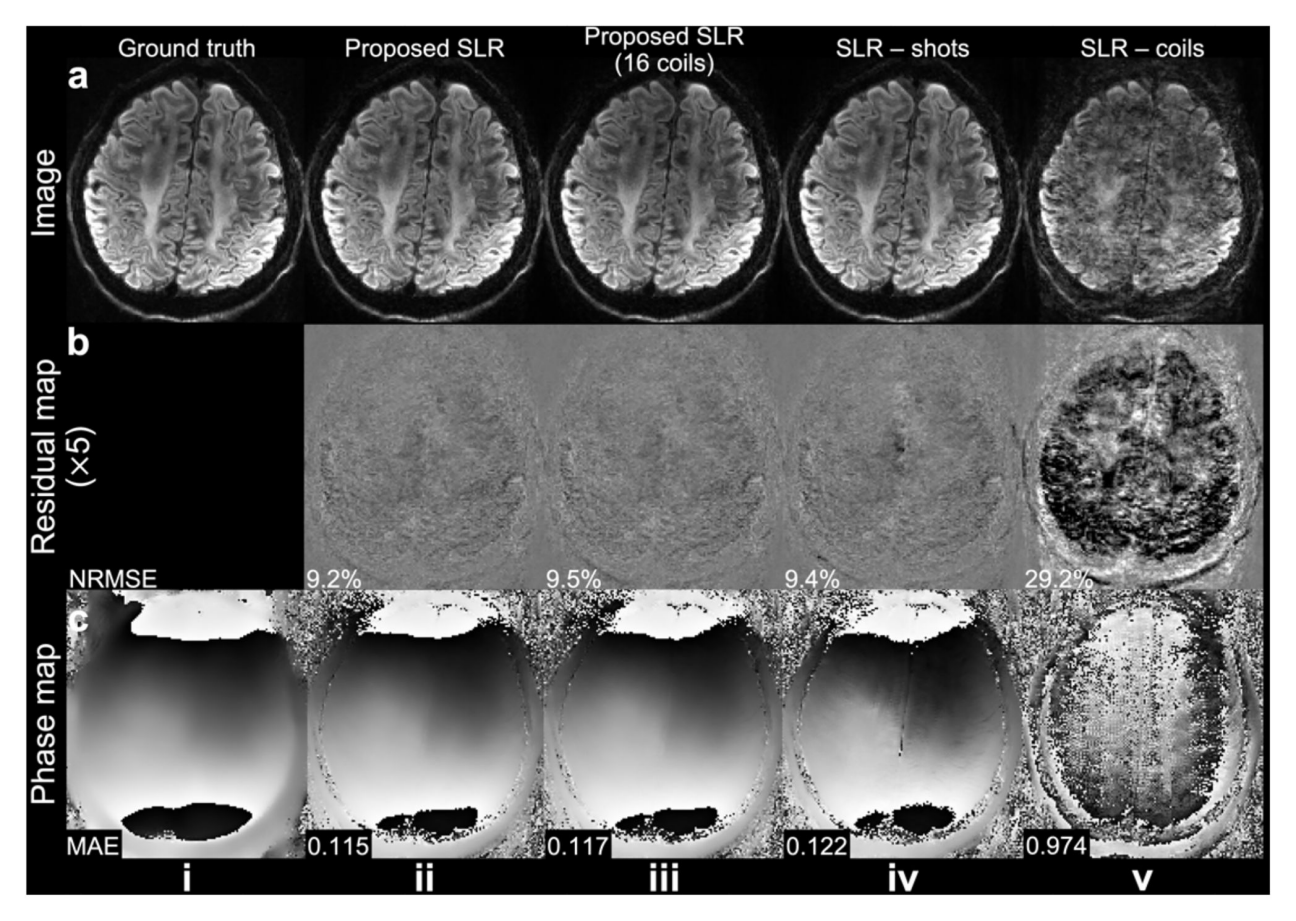
Comparison of different reconstruction configurations. Reconstruction of retrospectively under-sampled data (a), residual maps to the ground truth (b), and estimated 2D phase maps (without filtering) of one shot (c) at 1.22 mm isotropic resolution from Simulation Evaluation (Exp. 1.5, a slab-boundary slice) are shown. The 2D phase maps are reconstructed using the proposed structured low-rank (SLR) (ii), the proposed SLR with data from 16 coils (selected from 32 coils) (iii), SLR only leveraging redundancy across shots, i.e., coil-combined SLR (SLR-shots, iv), and SLR only leveraging redundancy across coils (SLR-coils, v). Note for SLR with data from 16 coils, the residual map and NRMSE are calculated based on the 16-coil fully sampled data.

The inclusion of both shot and coil dimensions benefits the SLR reconstruction (Fig. 9). Without leveraging shared information across shots, the SLR fails to estimate a reliable 2D phase map due to the high under-sampling factor of the self-navigation points for each shot (see Fig. 2d), and the image reconstruction is contaminated by the phase error (Fig. 9, v). Utilizing the redundancy across shots substantially benefits the SLR (Fig. 9, iv), while exploiting both shot and coil dimensions further improves the phase estimation accuracy, leading to a better image reconstruction (Fig. 9, ii). Notably, our method demonstrates robustness when using only 16-coil data (Fig. 9, iii), highlighting its compatibility with receive RF coils consisting of fewer channels.

**Fig. 10:**
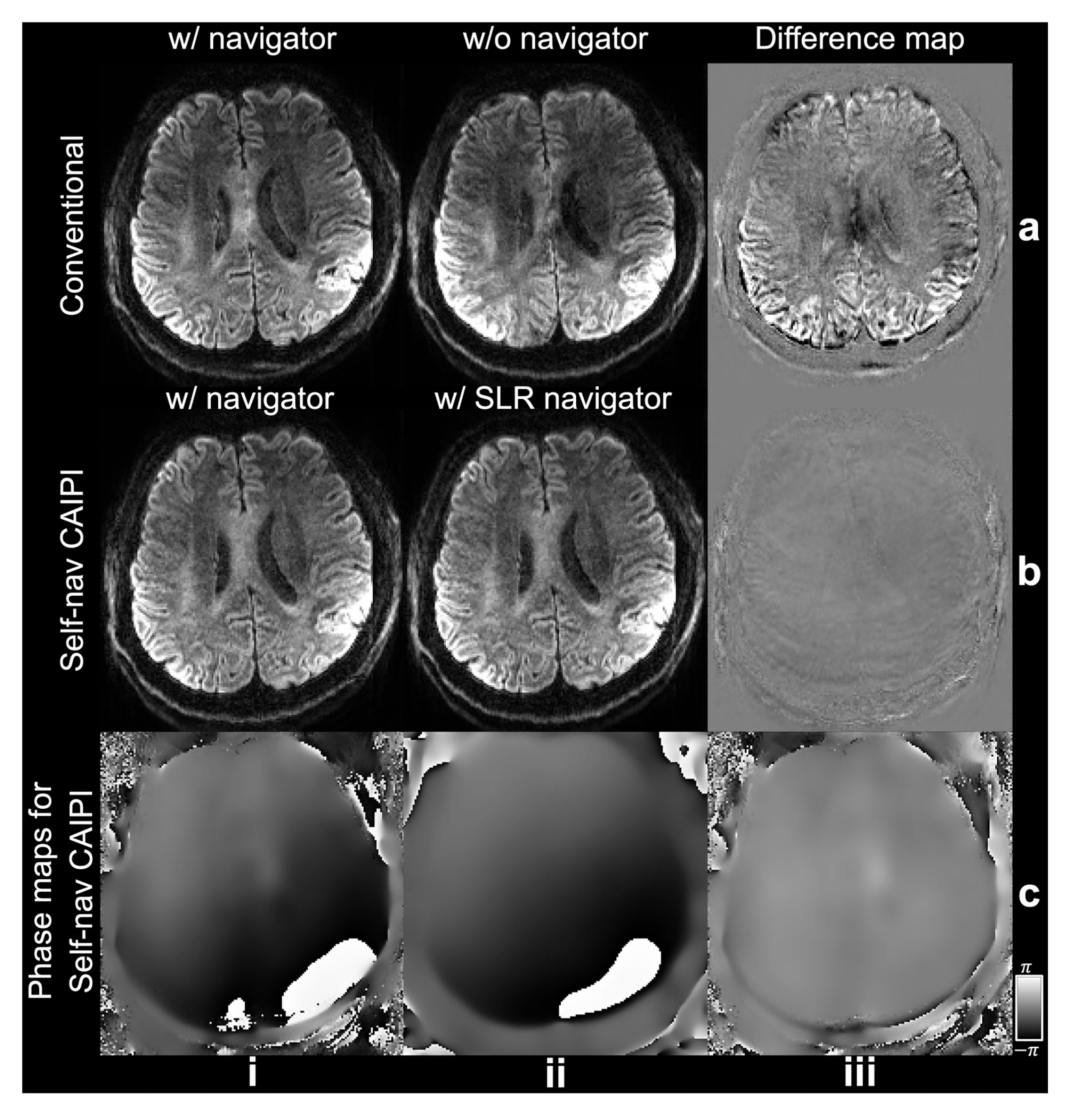
Reconstruction of prospectively acquired data. In-vivo diffusion weighted (diffusion encoding along the slice selection direction) images at 1.09 mm isotropic resolution of a representative subject from Exp. 2 are shown. Reconstruction using conventional rectangular sampling with (a, i) and without navigator (a, ii), and using Self-nav CAIPI sampling with explicitly acquired (b, i) and SLR estimated navigator (b, ii) are compared. The difference maps (iii) between i and ii are also presented. Additionally, the explicitly acquired and SLR estimated navigator phase map of a representative shot of Self-nav CAIPI sampling, along with their difference, are displayed (c).

Our method works well on prospectively acquired in-vivo data (Fig. 10). While the conventional method exhibits significant image artifacts when navigators are not acquired (Fig. 10a, ii, iii), our proposed self-navigated method achieves similar image reconstruction results using either navigator phase maps or SLR estimated phase maps (Fig. 10b). Notably, the navigator acquired phase map and estimated phase map exhibit high consistency (Fig. 10c).

**Fig. 11:**
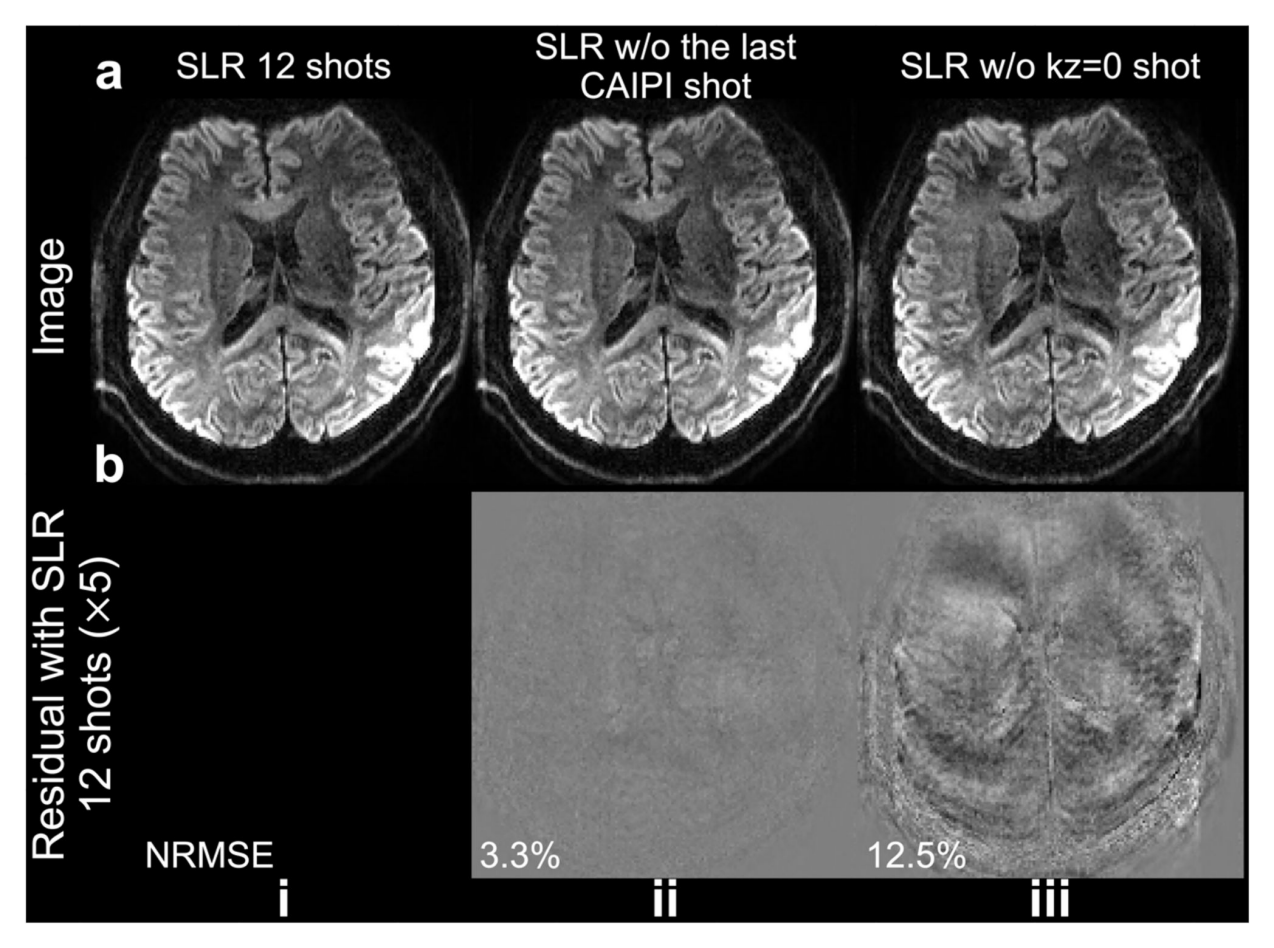
Impact of the kz=0 shot in the in-vivo experiment. In-vivo diffusion weighted (diffusion encoding along the slice selection direction) images acquired using Self-nav CAIPI sampling at 1.09 mm isotropic resolution of a representative subject from Exp. 2 are shown (a). Images reconstructed using the proposed structured low-rank (SLR) using data from all 12 shots (i), data excluding the last CAIPI shot (ii), and data excluding the kz=0 shot (iii) are compared, with residual maps (b) showing the difference with the 12-shot reconstruction.

The impact of the kz=0 traversal shot on reconstruction is notably evident in the in-vivo experiment (Fig. 11). Comparing reconstructions using the same amount of data from 11 shots, excluding the last CAIPI shot leads to a more accurate reconstruction (Fig. 11, ii) compared to exclusion of the kz=0 shot (Fig. 11, iii), indicating the importance of the kz=0 shot for precise 2D phase map estimation. Remarkably, removal of the last CAIPI shot does not significantly compromise image quality (Fig. 11, ii) thanks to the sampling optimization based on the greedy search framework.

**Fig. 12:**
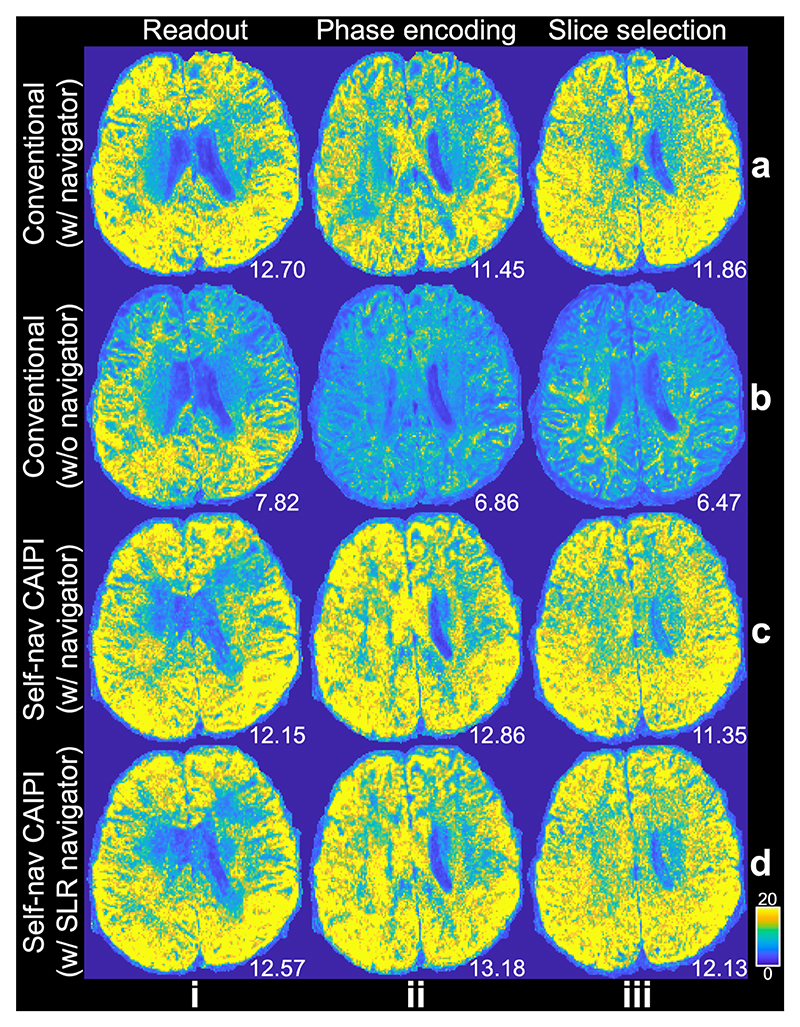
SNR maps of different diffusion encoding directions. The SNR maps for diffusion-weighted images (1.09 mm isotropic resolution) along readout (i), phase encoding (ii), and slice selection (iii) diffusion encoding directions from conventional and Self-nav CAIPI acquisition with the same TR of a representative subject from Exp. 2 are demonstrated. Four acquisition and reconstruction methods are compared: (a) conventional rectangular sampling and reconstruction with navigator phase maps. (b) conventional rectangular sampling and reconstruction without phase error correction. (c) Self-nav CAIPI acquisition and reconstruction with navigator phase maps. (d) Self-nav CAIPI acquisition and reconstruction with structured low-rank estimated phase maps (d). The mean SNR calculated within a brain mask for this subject is listed.

Our proposed method exhibits robust performance along different diffusion encoding directions (Fig. 12). When acquired with the same TR, the self-navigated method (Fig. 12d) produces SNR values comparable to the conventional navigated method (Fig. 12a), even along the slice selection diffusion encoding direction (Fig. 12, iii) where motion-induced phase variance is most significant [7]. Notably, the SNR values obtained from our self-navigated method are substantially higher than those from conventional sampling without phase error correction and even marginally higher than those from navigated Self-nav CAIPI sampling. This could be attributed to the higher resolution of SLR reconstructed phase maps compared to navigators (64 phase encoding lines acquired) which may fail to capture high-frequency phase changes in certain cases (i.e., larger motions). The group-level SNR values for three subjects (Exp. 2, Table II) are consistent with the findings depicted in Fig. 12.

**Fig. 13:**
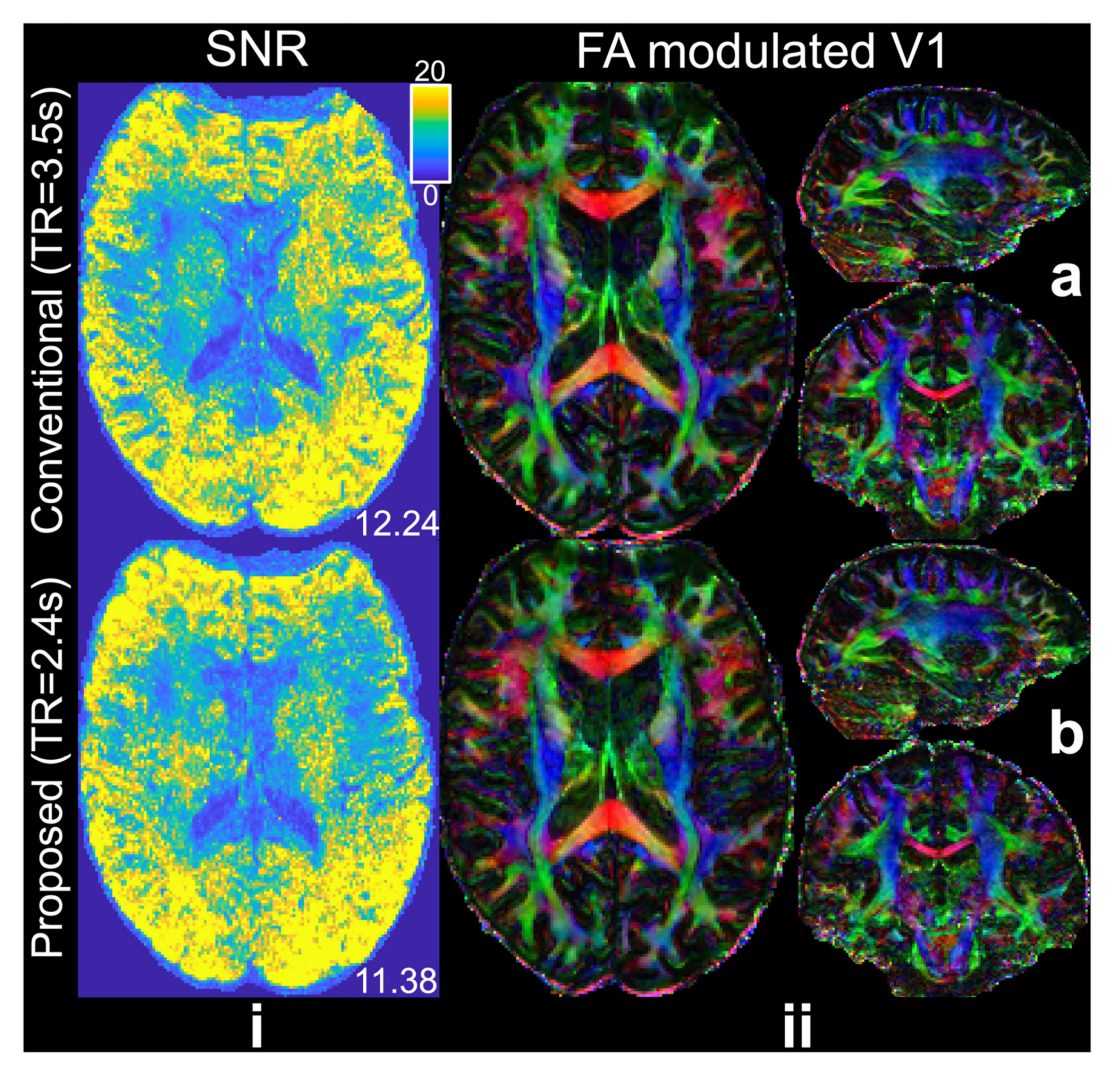
SNR efficiency and DTI comparisons. The comparison of SNR maps (Exp. 3, i) and DTI results (Exp. 4, ii, 1.09 mm isotropic resolution) of a representative subject is presented. The SNR maps and DTI results are obtained using the conventional navigated method (a) and the proposed self-navigated method (b). The whole-brain SNR values (calculated within a brain mask) for this subject are listed. Notably, the proposed method has a shorter TR by eliminating navigator acquisition.

Removing the navigator acquisition can shorten TR, leading to an improved SNR efficiency. With the TR reduction, the SNR obtained from the self-navigated method is slightly lower compared to the conventional navigated method (Fig. 13, i), as would be expected due to the reduced T1 recovery. For the four subjects scanned in Exp. 3, the group-level SNR values (mean ± std) are 11.70 ± 0.74 and 11.18 ± 0.41 for the conventional and proposed method, respectively. In terms of SNR efficiency, the group-level results for the conventional and proposed methods are 6.25 ± 0.39 and 7.22 ± 0.26, respectively. The self-navigated method improves SNR efficiency by 15.5% compared to the conventional method.

The self-navigated method enables high-quality DTI within a shorter scan time (Fig. 13, ii). Our method obtained the DTI maps within 8.6 minutes (Fig. 13b, ii), with comparable SNR to the conventional method requiring 12.6 minutes (Fig. 13a, ii). By eliminating the navigator acquisition, we save 31.7% of the total scan time without noticeable loss in image quality.

**Fig. 14:**
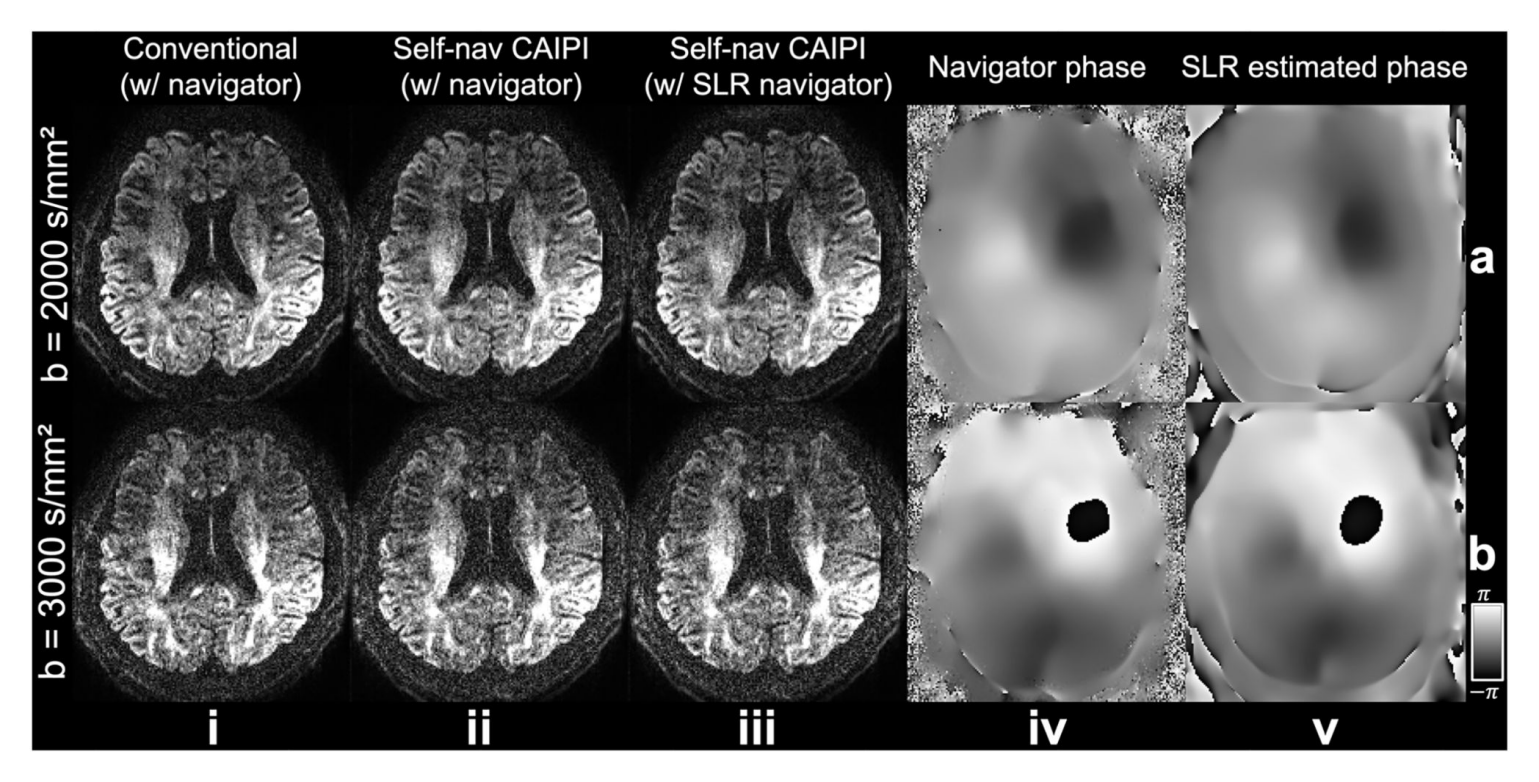
Results at high b-values. Diffusion-weighted images (1.09 mm isotropic resolution, diffusion encoding along readout) reconstructed using conventional sampling with navigator (i), Self-nav CAIPI sampling with navigator (ii), and Self-nav CAIPI sampling with structured low-rank estimated phase maps (iii) at b=2000 s/mm^2^ (a) and 3000 s/mm^2^ (b) from Exp. 5 are shown. The phase maps of one representative shot for Self-nav CAIPI sampling from the navigator (iv) and the proposed structured low-rank (SLR) reconstruction (v) are also demonstrated.

Fig. 14 demonstrates the performance of our method at higher b-values, a scenario where the diffusion data have lower SNR and are more sensitive to motion-induced phase errors. The Self-nav CAIPI sampling with SLR estimated phase maps (Fig. 14, iii) produces similar results to conventional rectangular sampling (Fig. 14, i) and Self-nav CAIPI sampling with navigator phase maps (Fig. 14, ii). The SLR estimated phase maps (Fig. 14, v) are highly similar to the navigator phase maps (Fig. 14, iv), even at high b-values (e.g., b=3000 s/mm^2^). This result demonstrates the robustness of the proposed sampling and reconstruction method at high b-values.

**Fig. 15:**
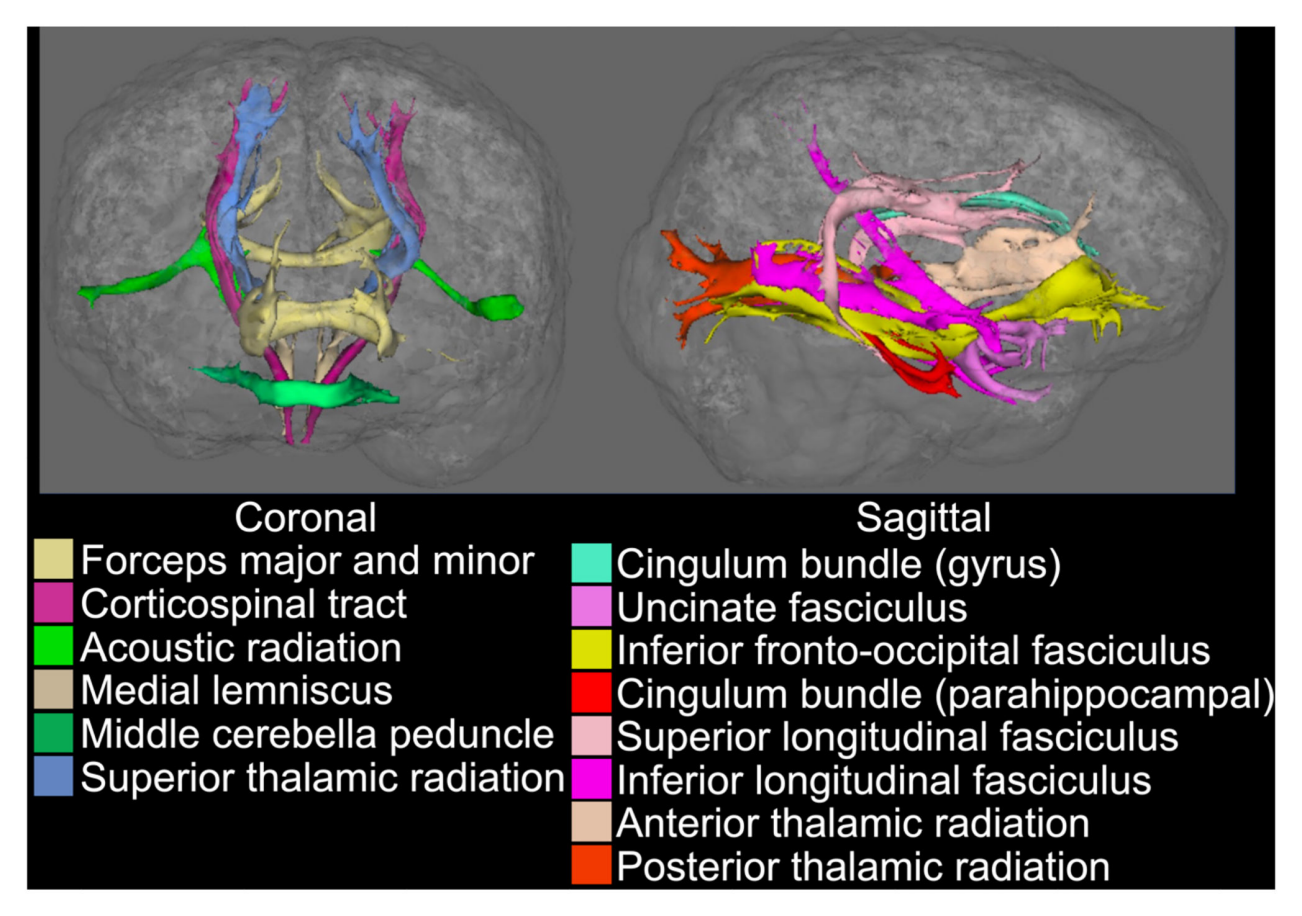
Tractography results. The binarized tractography (threshold: 0.01) results obtained from 48-direction diffusion data at 1.09 mm isotropic resolution using our proposed method from Exp. 6 are presented. Fourteen tracts are illustrated in coronal and sagittal views.

Fig. 15 demonstrates whole-brain tractography results using the proposed self-navigated method, with 14 tracts illustrated in coronal and sagittal views. The data acquired with the proposed method support the delineation of major fiber bundles.

## V Discussion

In this work, we propose a novel acquisition and reconstruction framework that extends 2D SLR-based phase correction [11, 12, 14] to 3D multi-slab diffusion MRI to eliminate the need for acquiring navigators. Each shot of our Self-nav CAIPI sampling intersects with the central kz=0 plane, providing self-navigation points. The sampling is optimized for overlapping, k-space gaps, and self-navigation performance using a greedy search algorithm. Self-nav CAIPI sampling allows us to leverage an SLR reconstruction method that exploits the redundancy across shots and coils to obtain a 2D phase map from self-navigation points for each shot. One shot traverses the entire kz=0 plane to provide accurate magnitude information, accelerating the convergence and improving the robustness of the reconstruction. In-vivo experiments from seven subjects validate the efficacy and robustness of our proposed method, which saves 31.7% of scan time by eliminating the navigator acquisition and achieves 15.5% higher SNR efficiency compared to conventional navigated 3D multi-slab imaging.

The SNR efficiency benefits of eliminating the navigator acquisition are more pronounced for high-field and high-resolution applications. High-field applications often face SAR constraints, necessitating longer TRs. In our baseline protocol, TR=3.5s is the minimal achievable TR to comply with SAR limits. Eliminating the 180° RF pulse for the navigator echo effectively reduces SAR and the required TR. On the other hand, higher resolution necessitates the acquisition of more slabs, and therefore a longer TR. For instance, a previous study which achieved 0.85 mm isotropic resolution used TR=3s at 3T [34]. In this case, eliminating navigator acquisition and shortening TR are expected to bring SNR efficiency benefits.

Our sampling design hinges on two critical considerations: optimizing for the multi-shot reconstruction and self-navigation performance (i.e., the reconstruction of kz=0 plane). We developed an optimization framework to determine the optimal sampling pattern using a shot-by-shot greedy search. Given the substantial parameter space (with $s_{k_{z}}\in [0,5],s_{k_{y}}\in [0,2]$, *s*_*y*_ ∈ [0,11]), conducting a global search across 11 shots would involve evaluating (6 × 3 × 12)^11^ ≈ 4.78 × 10^25^ sampling patterns. Our greedy search approach significantly reduces the search space to 6 × 3 × 12 × 11 = 2376 while achieving satisfactory reconstruction performance. Additionally, the uniform and random samplings may benefit compressed sensing reconstruction methods [19], indicating that adding tailored constraints in Eq. 22 may further enhance performance. Furthermore, our sampling strategy holds potential for additional acceleration by acquiring fewer shots (i.e., $N_{shot\;}<N_{k_{z}}$) without significant image quality loss (Fig. 11), as the greedy search-based sampling optimization ensures efficient k-space coverage even with fewer shots. Recent studies have similarly demonstrated that CAIPI sampling optimization can facilitate accelerated 3D multi-slab diffusion MRI [6, 35], supporting its benefits in under-sampled diffusion MRI reconstruction.

Our alternation in the sampling pattern is not expected to introduce significant addition geometric distortions or blurring compared to the conventional sampling. In our periodic sampling, the accumulations of B0 induced phase and T2* decay along the slice direction is effectively counteracted during the kz-blip-up and kz-blip-down steps (Fig. 2d), thereby minimizing additional geometric distortions and blurring along the slice direction. Consequently, our prospectively acquired in-vivo data using Self-nav CAIPI exhibit no significant additional distortions or blurring when compared to the conventional sampling (Figs. 10, 13, 14).

Due to the limited number of self-navigation points, the acceleration factor for kz=0 reconstruction is extremely high (R=18 or 36, Fig. 2d). The substantial under-sampling factor necessitates the utilization of shared information from other shots for accurate reconstruction (Fig. 9, v). Incorporating the coil dimension exploits the inherent low-rankness between coils due to the smooth nature of coil sensitivity, thereby benefiting parallel imaging reconstruction (Fig. 9, iv) [21]. The impact is expected to be more pronounced for low-SNR in-vivo data. The shot traversing kz=0 provides an accurate magnitude estimation, reducing the unknowns from the complex image to only the phase image. This is proved beneficial for accelerating the convergence and improving the robustness of the reconstruction (Figs. 7, 11). The optimized self-navigation performance (i.e., *d*_*i*_ in Eq. 11) ensures that each shot contains sufficient low-frequency information to produce a reliable phase map. These attributes enable robust reconstruction of 2D phase maps from limited data, even with low-SNR (e.g., high b-values, Fig. 14).

Our method outperforms the previous self-navigation method [9] (Fig. 8), which used conventional sampling and extracted a phase map from each kz plane. We found our data necessitated a stronger filter than the original work to suppress the high-frequency noise, presumably due to the larger number of kz planes per slab used in our work (12 kz vs. 8 kz). The estimated phase maps are over-smoothed and fail to accurately capture the phase variation (Fig. 8c, ii). This also highlights the intrinsic challenge to extract smooth and accurate phase maps from peripheral kz planes that encode high-frequency information, which might limit the performance and robustness of the previous method.

The SLR estimated phase maps demonstrate slightly better reconstruction performance than navigator-acquired phase maps in prospective in-vivo experiments (Fig. 8, Table II). However, in Exp. 1, the structured low-rank reconstruction exhibits slightly higher NRMSE compared to navigated reconstruction (Fig. 4). This is presumably because in Exp. 1 the navigator phase maps are ground truth phase maps that were retrospectively added to phase error-free data. In prospective acquisitions, navigators contain some measurement error due to the extended echo time of their acquisition and the associated SNR loss. It has been demonstrated that reducing the resolution along the readout direction and decreasing the acceleration factor along the phase encoding direction can effectively mitigate such SNR loss and measurement error [36, 37]. Furthermore, compared to the conventional method where the navigators are acquired with lower resolutions, our SLR reconstructed phase maps have the same resolution as the images, which might be helpful in capturing motion-induced phase variations more accurately.

The inclusion of both shot and coil dimensions contributes to the substantial size of the Hankel matrix $H(\hat{x})$ (Fig. 3), making the current reconstruction time-consuming. Each iteration of the ADMM takes approximately 8 minutes on a 2.9 GHz Quad-Core Intel Core i7 CPU, resulting in a reconstruction time of ~7 hours for a 3D slab on a single CPU. The inclusion of the kz=0 traversing shot and the magnitude constraint $||\hat{x}-Fm^{\prime }\Phi^{k-1}|{|}_{2}^{2}$ may help reduce the number of iterations without compromising the reconstructed phase map quality (Fig. 7c). Using the coil-combined SLR (i.e., “SLR – shots” in Fig. 9, iv) formulation can also effectively shorten the computation time of each iteration to 3 minutes with a slight performance compromise. Model-based deep learning reconstruction approaches [14] may also offer a promising avenue for accelerating the reconstruction process.

It is also worth noting that our method primarily addresses phase error induced by physiological motions such as respiration and cardiac pulsations. Additional considerations are necessary to address larger-scale motions. For example, inter-shot bulk motions violate the assumption of the SLR that the underlying kz=0 magnitude images are consistent across different shots. Recent advances [38] which integrate rigid motion compensation into SLR hold promises in addressing such motions without acquiring navigators. However, for large-scale motion particularly along the slice direction, the 2D phase maps might be no longer sufficient for accurately capturing the motion. In this case, data rejection and reacquisition [39] or prospective motion correction methods [40] should be considered.

## VI Conclusion

We present a novel acquisition and reconstruction framework that eliminates the requirement for navigator acquisition in high-resolution 3D multi-slab diffusion MRI. It effectively shortens the TR and reduces SAR, enhancing scan efficiency and safety, and permitting the use of better RF pulses. The effectiveness of our method is evident in high-fidelity fast DTI and tractography and can be explored in more applications.

## Figures and Tables

**Table I:** ACQUISITION PARAMETERS

Exp.#	Voxel size (mm^3^)	TE1 (imaging) / TE2 (navigator) / TR(ms)^a^	b-value (s/mm^2^)	#b=0 /DWI^b^	Sampling^c^	Navigator	T_acq_ (min:sec)
1A	1.22	82/142/2000	1000	1/3	Conventional	Yes	1:36
1B	1.09	89/157/3500	1000	1/3	Conventional	Yes	2:48
2A	1.09	65/133/3500	1000	0/36	Conventional	Yes	25:12
2B	1.09	65/133/3500	1000	0/36	Self-nav CAIPI	Yes	25:12
3A	1.09	65/133/3500	1000	0/12	Conventional	Yes	8:24
3B	1.09	64/-/2400	1000	0/12	Self-nav CAIPI	No	5:46
4A	1.09	65/133/3500	1000	2/16	Conventional	Yes	12:36
4B	1.09	64/-/2400	1000	2/16	Self-nav CAIPI	No	8:38
5A	1.09	72/140/3500	2000	0/1	Conventional	Yes	0:42
5B	1.09	72/140/3500	2000	0/1	Self-nav CAIPI	Yes	0:42
5C	1.09	78/146/3500	3000	0/1	Conventional	Yes	0:42
5D	1.09	78/146/3500	3000	0/1	Self-nav CAIPI	Yes	0:42
6	1.09	64/-/2400	1000	6/48	Self-nav CAIPI	No	25:55

a 3/4 partial Fourier along phase encoding direction was applied for Exp. 2-6. The TRs of Exp. 2-6 were the shortest achievable TRs due to SAR restriction.

b The number of DWIs refers to the number of scans with different encoding for each experiment. In Exp. 1, three Δky shift was acquired with the same diffusion encoding. In Exp. 2, 12 repetitions along three orthogonal diffusion encoding directions were obtained for SNR evaluation. In Exp. 3, 12 repetitions were acquired with a single diffusion encoding direction along readout.

c The Self-nav CAIPI samplings for 1.22 mm and 1.09 mm are illustrated in Fig. 2e and f respectively. All b=0 data were acquired with the conventional rectangular sampling.

**Table II:** GROUP-LEVEL SNR VALUES ALONG DIFFERENT DIFFUSION ENCODING DIRECTIONS

	Readout	Phase encoding	Slice selection
Convectional (w/ navigator)	11.66±1.23	11.33±0.37	**11.65±0.38**
Conventional (w/o navigator)	7.37±0.53	6.91±0.13	6.14±0.33
Self-nav CAIPI (w/ navigator)	11.46±0.72	12.46±0.45	10.57±0.73
Self-nav CAIPI (w/ SLR navigator)	**11.95±0.60**	**13.04±0.16**	11.43±0.81

The group level SNR (mean ± std) for different diffusion encoding directions for different acquisition and reconstruction methods of four subjects in Exp. 2 are compared. The highest SNR for each diffusion direction is marked in bold.

## References

[1] Miller KL (2011). Diffusion imaging of whole, post-mortem human brains on a clinical MRI scanner. Neuroimage.

[2] McNab JA (2013). Surface based analysis of diffusion orientation for identifying architectonic domains in the in vivo human cortex. Neuroimage.

[3] Frost R, Miller KL, Tijssen RH, Porter DA, Jezzard P (2014). 3D Multi-slab diffusion-weighted readout-segmented EPI with real-time cardiac-reordered k-space acquisition. Magnetic resonance in medicine.

[4] Wu W (2016). High-resolution diffusion MRI at 7T using a three-dimensional multi-slab acquisition. NeuroImage.

[5] Engström M, Skare S (2013). Diffusion-weighted 3D multislab echo planar imaging for high signal-to-noise ratio efficiency and isotropic image resolution. Magnetic resonance in medicine.

[6] Li Z (2023). Sampling strategies and integrated reconstruction for reducing distortion and boundary slice aliasing in high-resolution 3D diffusion MRI. Magnetic Resonance in Medicine.

[7] Miller KL, Pauly JM (2003). Nonlinear phase correction for navigated diffusion imaging. Magnetic Resonance in Medicine: An Official Journal of the International Society for Magnetic Resonance in Medicine.

[8] Butts K, de Crespigny A, Pauly JM, Moseley M (1996). Diffusion-weighted interleaved echo-planar imaging with a pair of orthogonal navigator echoes. Magnetic resonance in medicine.

[9] Moeller S (2020). Self-navigation for 3D multishot EPI with data-reference. Magnetic resonance in medicine.

[10] Chen N-k, Guidon A, Chang H-C, Song AW (2013). A robust multi-shot scan strategy for high-resolution diffusion weighted MRI enabled by multiplexed sensitivity-encoding (MUSE). Neuroimage.

[11] Mani M, Jacob M, Kelley D, Magnotta V (2017). Multi - shot sensitivity-encoded diffusion data recovery using structured low-rank matrix completion (MUSSELS). Magnetic resonance in medicine.

[12] Mani M, Aggarwal HK, Magnotta V, Jacob M (2020). Improved MUSSELS reconstruction for high-resolution multi-shot diffusion weighted imaging. Magnetic resonance in medicine.

[13] Markovsky I (2008). Structured low-rank approximation and its applications. Automatica.

[14] Aggarwal HK, Mani MP, Jacob M (2019). MoDL-MUSSELS: model-based deep learning for multishot sensitivity-encoded diffusion MRI. IEEE transactions on medical imaging.

[15] Liao C (2021). Distortion-free, high-isotropic-resolution diffusion MRI with gSlider BUDA-EPI and multicoil dynamic B0 shimming. Magnetic resonance in medicine.

[16] Liao C (2023). High-fidelity mesoscale in-vivo diffusion MRI through gSlider-BUDA and circular EPI with S-LORAKS reconstruction. NeuroImage.

[17] Breuer FA (2006). Controlled aliasing in volumetric parallel imaging (2D CAIPIRINHA). Magnetic Resonance in Medicine: An Official Journal of the International Society for Magnetic Resonance in Medicine.

[18] Lustig M, Pauly JM (2010). SPIRiT: iterative self-consistent parallel imaging reconstruction from arbitrary k-space. Magnetic resonance in medicine.

[19] Seeger M, Nickisch H, Pohmann R, Schölkopf B (2010). Optimization of k-space trajectories for compressed sensing by Bayesian experimental design. Magnetic Resonance in Medicine: An Official Journal of the International Society for Magnetic Resonance in Medicine.

[20] Murphy M, Alley M, Demmel J, Keutzer K, Vasanawala S, Lustig M (2012). Fast $\ell_1 $-SPIRiT compressed sensing parallel imaging MRI: scalable parallel implementation and clinically feasible runtime. IEEE transactions on medical imaging.

[21] Shin PJ (2014). Calibrationless parallel imaging reconstruction based on structured low - rank matrix completion. Magnetic resonance in medicine.

[22] Griswold MA (2002). Generalized autocalibrating partially parallel acquisitions (GRAPPA). Magnetic Resonance in Medicine: An Official Journal of the International Society for Magnetic Resonance in Medicine.

[23] Boyd S, Parikh N, Chu E, Peleato B, Eckstein J (2011). Distributed optimization and statistical learning via the alternating direction method of multipliers. Foundations and Trends® in Machine learning.

[24] Chen X, Wu W, Chiew M (2023). Improving robustness of 3D multi-shot EPI by structured low-rank reconstruction of segmented CAIPI sampling for fMRI at 7T. NeuroImage.

[25] Uecker M (2014). ESPIRiT—an eigenvalue approach to autocalibrating parallel MRI: where SENSE meets GRAPPA. Magnetic resonance in medicine.

[26] Zhang T, Pauly JM, Vasanawala SS, Lustig M (2013). Coil compression for accelerated imaging with Cartesian sampling. Magnetic resonance in medicine.

[27] Jenkinson M, Beckmann CF, Behrens TE, Woolrich MW, Smith SM (2012). Fsl. Neuroimage.

[28] Jenkinson M, Smith S (2001). A global optimisation method for robust affine registration of brain images. Medical image analysis.

[29] Wu W, Koopmans PJ, Frost R, Miller KL (2016). Reducing slab boundary artifacts in three-dimensional multislab diffusion MRI using nonlinear inversion for slab profile encoding (NPEN). Magnetic resonance in medicine.

[30] Bautista T, O’Muircheartaigh Hajnal J, Donald TJ (2021). Removal of Gibbs ringing artefacts for 3D acquisitions using subvoxel shifts. Proc Int Soc Magn Reson Med.

[31] Andersson JL, Skare S, Ashburner J (2003). How to correct susceptibility distortions in spin-echo echo-planar images: application to diffusion tensor imaging. Neuroimage.

[32] Andersson JL, Sotiropoulos SN (2016). An integrated approach to correction for off-resonance effects and subject movement in diffusion MR imaging. Neuroimage.

[33] De Groot M (2013). Improving alignment in tract-based spatial statistics: evaluation and optimization of image registration. Neuroimage.

[34] Chang H-C (2015). Human brain diffusion tensor imaging at submillimeter isotropic resolution on a 3 Tesla clinical MRI scanner. Neuroimage.

[35] Lee C-Y, Mani M (2024). 2D CAIPI accelerated 3D multi-slab diffusion weighted EPI combined with qModeL reconstruction for fast high resolution microstructure imaging. Magnetic Resonance Imaging.

[36] Dai E (2018). The effects of navigator distortion and noise level on interleaved EPI DWI reconstruction: a comparison between image-and k-space-based method. Magnetic Resonance in Medicine.

[37] Dai E, Liu S, Guo H (2021). High-resolution whole-brain diffusion MRI at 3T using simultaneous multi-slab (SMSlab) acquisition. NeuroImage.

[38] Chen X, Wu W, Chiew M (2024). Motion compensated structured low-rank reconstruction for 3D multi-shot EPI. Magnetic Resonance in Medicine.

[39] Porter DA, Heidemann RM (2009). High resolution diffusion-weighted imaging using readout - segmented echo - planar imaging, parallel imaging and a two - dimensional navigator - based reacquisition. Magnetic Resonance in Medicine: An Official Journal of the International Society for Magnetic Resonance in Medicine.

[40] Maclaren J, Herbst M, Speck O, Zaitsev M (2013). Prospective motion correction in brain imaging: a review. Magnetic resonance in medicine.

